# Abnormal lipid metabolism and atherosclerosis: a new perspective on organelle function regulation and ferroptosis

**DOI:** 10.3389/fimmu.2025.1642984

**Published:** 2025-08-14

**Authors:** Xize Wu, Yuxi Huang, Jiaqi Ren, Xue Pan, Qiuying Wu, Qicheng Cai, Ruiying Wang, Teng Feng, Shan Gao, Bo Wang, Meijia Cheng, Yue Li, Lihong Gong

**Affiliations:** ^1^ The First Clinical College, Liaoning University of Traditional Chinese Medicine, Shenyang, China; ^2^ College of Traditional Chinese Medicine, Dazhou Vocational College of Chinese Medicine, Dazhou, China; ^3^ Department of Cardiology, Affiliated Hospital of Liaoning University of Traditional Chinese Medicine, Shenyang, China

**Keywords:** atherosclerosis, ferroptosis, bioinformatics, machine learning, mitochondrion, lysosome

## Abstract

**Background:**

Atherosclerosis (AS), characterized by lipid accumulation, contributes significantly to global cardiovascular morbidity. Ferroptosis, an iron-dependent form of cell death triggered by lipid peroxidation, is emerging as a critical player in AS progression. Therefore, our study seeks to elucidate the intricate mechanisms of ferroptosis within the lipid metabolism pathway in AS.

**Methods:**

Differentially expressed genes were identified from the GSE100927 dataset, subsequently isolating AS lipid metabolism-related ferroptosis genes (ASLMRFeGs). Unsupervised cluster analysis was performed on AS samples to identify molecular clusters. WGCNA was performed to uncover module Hub genes. Multiple machine learning models (LASSO, SVM-RFE, RF) were applied to screen Hub genes. Experimental validation was performed by ox-LDL-induced HUVECs and RAW 264.7 cells. Single-cell data analyzes the gene structure and gene expression status of individual cells.

**Results:**

Six ASLMRFeGs (CTSB, CYBB, DPP4, HILPDA, HMOX1, IL1B) alter the immune microenvironment in AS. AS samples were stratified into two molecular clusters, exhibiting significant variations in inflammation and immune responses. Enrichment analysis of the 225 module Hub genes showed close association with inflammation, immune responses, cytoskeleton organization, and various organelles. Machine learning identified four candidate Hub genes (TYROBP, CSF1R, LCP2, C1QA). *In vitro* experiments showed that dysregulated lipid metabolism promotes ferroptosis, and inhibition of ferroptosis improves mitochondrial and lysosomal dysfunction and suppresses endoplasmic reticulum stress. Ferrostatin-1, an ferroptosis inhibitor, attenuated the ox-LDL-induced upregulation of CYBB, HMOX1, IL1B, TYROBP, and CSF1R genes. A nomogram for predicting AS risk was constructed incorporating the expression levels of these five validated Hub genes. Single-cell data analysis results suggested that these genes were highly expressed in foam cells, inflammatory macrophages, smooth muscle cells, and helper T cells.

**Conclusion:**

In AS, abnormal lipid metabolism may drive ferroptosis via key regulatory genes (CYBB, HMOX1, IL1B, TYROBP, CSF1R), while also reshaping the immune microenvironment, potentially through the modulation of organelle function.

## 1 Introduction

Atherosclerosis (AS) is a chronic inflammatory condition that significantly impacts the vascular wall, primarily due to disruptions in lipid metabolism, vascular endothelial dysfunction, and inflammation (1, 2). AS serves as the foundational pathology underlying various cardiovascular diseases (CVDs), including ischemic heart disease and stroke, which continue to be leading causes of mortality in China and contribute substantially to global morbidity and mortality, with an alarming upward trend year by year (3).

The concept of “ferroptosis”, a recently unveiled form of cell death characterized by its iron-dependent accumulation of lipid peroxides, is central to our study. Ferroptosis represents an oxidative mode of cell death resulting from a pronounced buildup of reactive oxygen species (ROS) consequent to a decline in intracellular antioxidant capacity (4). Multiple mechanistic revelations underscore the pivotal role of ferroptosis in AS progression and plaque formation. These mechanisms encompass the induction of endothelial cell damage, activation of macrophage-mediated inflammatory responses, promotion of foam cell generation, and facilitation of the proliferation and migration of vascular smooth muscle cells (5). However, herein lies a crucial research gap. While the relationship between lipid metabolism and AS has been extensively studied (6), the intricate interplay between lipid metabolism and ferroptosis within the context of AS remains relatively uncharted territory. This study aims to bridge this gap by providing novel insights into the pathogenesis of AS and potential therapeutic avenues.

To delve into the mechanisms that underlie lipid metabolism in AS and ferroptosis, our study embarked on a comprehensive exploration. It commenced with a differential expression analysis, aimed at pinpointing AS lipid metabolism-related ferroptosis genes (ASLMRFeGs) and unraveling the immune profiles by juxtaposing control and AS samples. Subsequently, unsupervised cluster analysis was executed on the AS specimens predicated on ASLMRFeGs, leading to the delineation of molecular clusters with inherent associations with lipid metabolism and ferroptosis. Furthermore, this analysis strived to discern disparities in gene expression, immune responses, and underlying biological processes inherent to these clusters. Following this, the weighted gene co-expression network analysis (WGCNA) approach came into play, facilitating the identification of module Hub genes. To uncover candidate Hub genes, multiple topology algorithms were harnessed. Leveraging these identified Hub genes, we embarked on constructing a series of predictive models via machine learning algorithms. The performance of these predictive models underwent rigorous validation, encompassing the deployment of a nomogram, calibration curves, decision curve analysis (DCA), and scrutiny across three external datasets. Lastly, we ventured into the realm of predicting potential therapeutic agents for AS, grounded in ASLMRFeGs and Hub genes. The objective of this study is to investigate, through a series of bioinformatics analyses, the key genes involved in ferroptosis triggered by abnormal lipid metabolism in the context of AS, as well as the underlying mechanisms and the impact of these genes on the immune microenvironment. Furthermore, the study includes *in vitro* experiments to validate the role of these key genes in ferroptosis and lipid metabolism disorders, and to assess the influence of cellular organelles on lipid metabolism anomalies and ferroptosis.

## 2 Materials and methods

### 2.1 Bioinformatics analysis

#### 2.1.1 Subjects and dataset acquisition

**Figure 1:**
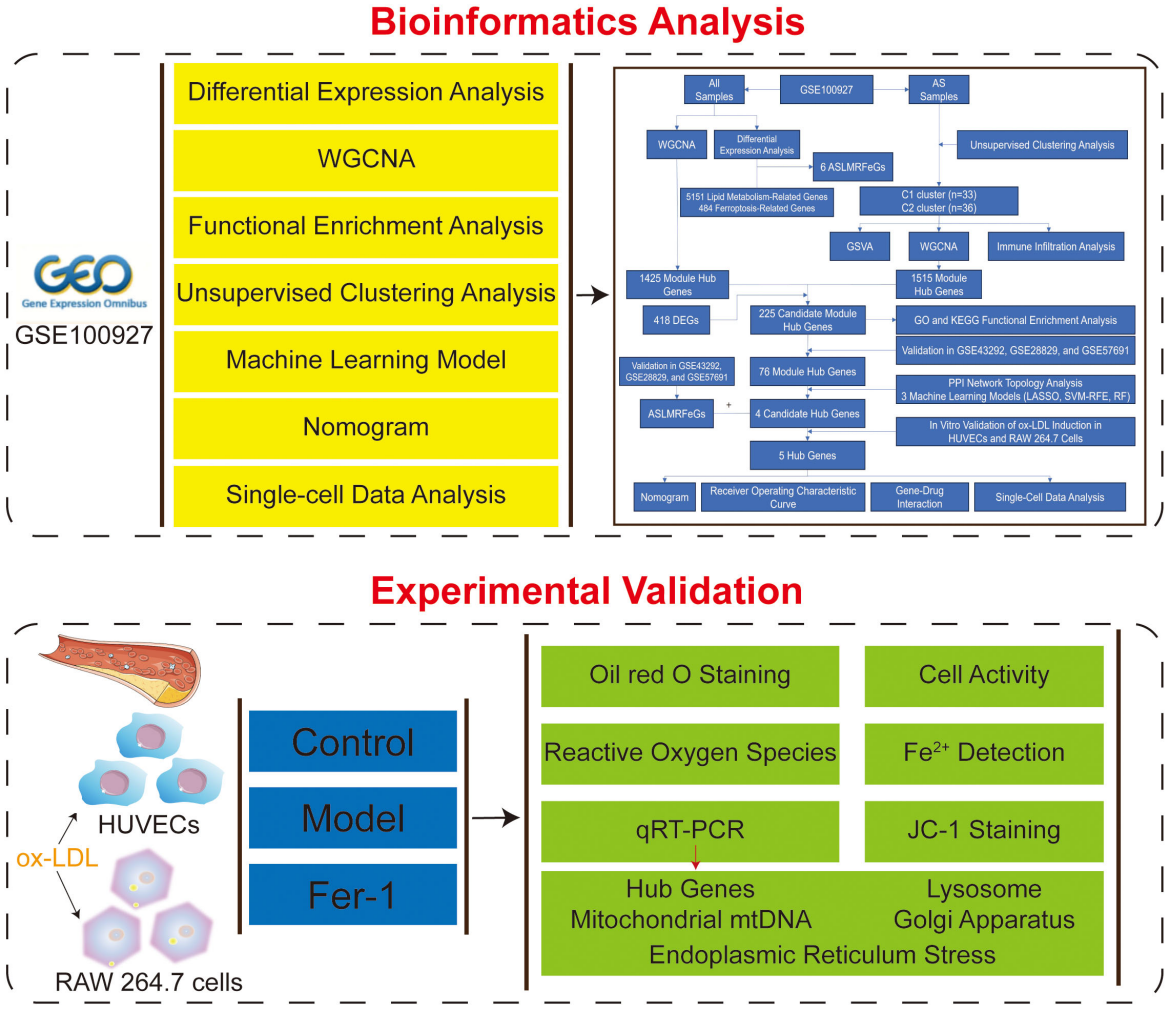
Flowchart of this study.

The entire study process is depicted in **Figure 1**. Five gene expression profiles (GSE100927, GSE43292, GSE28829, GSE57691, and GSE159677) related to AS were retrieved from the Gene Expression Omnibus (GEO, https://www.ncbi.nlm.nih.gov/geo/) database using the keywords “atherosclerosis” with species limited to “Homo sapiens.” The GSE100927 dataset (GPL17077) includes 35 healthy control arteries and 69 atherosclerotic diseased arteries, totaling 104 samples that served as the training set (7). The datasets GSE43292 (GPL6244), GSE28829 (GPL570), and GSE57691 (GPL10558) were used as validation sets, with the GSE43292 dataset including 32 healthy control arteries and 32 atherosclerotic diseased arteries, totaling 64 samples (8); the GSE28829 dataset including 13 early atherosclerotic plaques and 16 advanced atherosclerotic plaques, totaling 29 samples (9); and the GSE57691 dataset including 10 control samples and 9 abdominal aortic samples from patients with abdominal aortic occlusive disease, totaling 19 samples (10). Lastly, the GSE159677 dataset (GPL18573) for single-cell data analysis includes 3 carotid atherosclerotic cores and 3 control samples (11).

#### 2.1.2 Identification of differentially expressed genes and ASLMRFeGs

The GSE100927 dataset was used to identify the DEGs associated with AS by comparing the disease and control groups using the “limma” R package, with the criteria set at adj.*P*<0.05 and |logFC|>1 for DEGs selection (12). Lipid metabolism-related genes (LMRGs) were obtained from the Molecular Signature Database (MsigDB, https://www.gsea-msigdb.org/) and supplemented with genes having a “relevance score” greater than the mean (4.1689) from the GeneCards (https://www.genecards.org/, accessed on March 15, 2025) database under the keyword “lipid metabolism”. Ferroptosis-related genes (FeRGs), including driver, suppressor, and marker genes, were obtained from the FerrDb V2 (http://www.zhounan.org/ferrdb/current/, accessed on March 15, 2025) database (13). The intersection of DEGs, LMRGs, and FeRGs yielded ASLMRFeGs.

#### 2.1.3 Functional enrichment analysis

Gene Ontology and Kyoto Encyclopedia of Genes and Genomes (KEGG) were performed using the DAVID (https://davidbioinformatics.nih.gov/home.jsp, accessed on March 15, 2025) database (*P*<0.05). Gene Set Enrichment Analysis (GSEA) was conducted via the “GSEA” and “clusterProfiler” R package (14). KEGG pathway gene sets (c2.cp.kegg.v7.4.symbols.gmt) were obtained from the MSigDB database. Genes were ranked in descending order based on logFC values of differentially expressed genes. GSEA was performed using the clusterProfiler R package with default weighted enrichment statistic calculation. Multiple hypothesis testing correction was applied using the Benjamini-Hochberg method, retaining significant pathways with FDR < 0.05. The most significantly enriched pathways (NES > 0 for upregulated, NES < 0 for downregulated) were visualized using enrichplot.

#### 2.1.4 Immune infiltration and correlation analysis

We performed immune cell quantification using the CIBERSORT deconvolution algorithm with the LM22 signature matrix (containing gene expression signatures of 22 human immune cell subtypes) (15). Data preprocessing involved automatic logarithmic transformation, quantile normalization, and Z-score normalization. The ν-support vector regression (ν-SVR) algorithm was implemented with parameter optimization (ν=0.25-0.75), model selection by root-mean-square error minimization, and non-negative constraint enforcement. To evaluate the reliability of our results, we performed a 1000 permutation test (perm=1000), retaining significant results with a *P* < 0.05. Differences between the two groups (C1 compared to C2 cluster, control compared to AS group) were compared using the Wilcox test, and the results were visualized using the “vioplot” R package (16). Subsequently, Spearman correlation analysis was employed to reveal the relationship between ASLMRFeGs and immune cells.

#### 2.1.5 Unsupervised clustering analysis

Unsupervised clustering analysis of AS samples based on ASLMRFeGs expression profiles was performed using the “ConsensusClusterPlus” R package (17). The AS samples were grouped by applying the k-means algorithm with 1000 iterations, k=9, reps=50, pItem=0.8, pFeature=1, clusterAlg=km, distance=euclidean. The appropriate number of clusters was determined based on the matrix heat map, cumulative distribution function (CDF) curve, consensus matrix, and consistent cluster score (>0.9).

#### 2.1.6 Gene set variation analysis

We performed pathway activity quantification using GSVA on normalized expression profiles with the “c2.cp.kegg.symbols.gmt” gene set (18). The expression matrix was de-duplicated using the avereps function and filtered to include only AS group samples. Single-sample pathway enrichment scores were calculated using the GSVA algorithm, normalized by min-max scaling. Differential pathway analysis between predefined clusters (C1 compared to C2 cluster) was performed using two-sample t-tests (adj.*P*<0.05). The final visualization highlighted the up- and down-regulated pathways with the most significant differences (|t-value| > 2).

#### 2.1.7 WGCNA

We performed co-expression network analysis using the WGCNA R package (19). We retained genes with expression standard deviations ranking in the top 25% of highly variable genes and excluded low-expression genes and low-quality samples. The Euclidean distance matrix of gene expression profiles was calculated, and a hierarchical clustering dendrogram was constructed using the average linkage method. A cut height of 80 was set to remove samples that were too distant from the central cluster. The pickSoftThreshold function was used to evaluate the scale-free topology fit, selecting the minimum β value (soft thresholding power) that ensured R² > 0.9 for scale-free network construction. A weighted adjacency matrix was constructed based on the Pearson correlation coefficient raised to the power of the chosen β, and the topological overlap matrix (TOM) was further calculated to reduce noise interference. Dynamic tree cutting clustering was performed with parameters set as follows: minModuleSize=100, deepSplit=2, mergeCutHeight=0.25, and modules were divided using the cutreeDynamic function, with highly correlated modules (cor > 0.75) merged using the mergeCloseModules function. The first principal component of each module was calculated using moduleEigengenes. Pearson correlation with phenotypic data was computed, and significance was adjusted using Student’s t-test. Genes with expression correlated with the target trait (GS) and module eigengenes (MM) were identified, with genes defined as hub genes if GS > 0.5 and MM > 0.8.

#### 2.1.8 Construction of Protein-Protein Interaction network and screening of candidate hub genes

To further narrow down the Hub genes, the module Hub genes were imported into the STRING database (https://www.string-db.org/, accessed on March 15, 2025), and “Homo sapiens” was selected as the species with high confidence (0.7) (20). The PPI network of modular Hub genes was constructed, and topological analysis was performed using the Cytoscape plugin “CytoHubba” to filter out the candidate Hub genes with degree centrality, betweenness centrality, and closeness centrality greater than the mean and construct the PPI network of candidate Hub genes (21).

#### 2.1.9 Construction of predictive model based on multiple machine learning methods

The Least Absolute Shrinkage and Selection Operator (LASSO) regression algorithm is a linear model that achieves feature selection and sparsity by introducing an L1 regularization term, effectively screening out important variables and reducing model complexity (22). Support Vector Machine-Recursive Feature Elimination (SVM-RFE) optimizes the feature set by recursively training SVM models and eliminating the features with the smallest weights (23). Random Forest (RF) is an ensemble learning method that assesses the importance and contribution of features by constructing multiple decision trees.

We utilized the “glmnet” R package to perform LASSO regression with a binomial distribution (family=binomial) and L1 regularization (alpha=1). The optimal lambda value was determined through 10-fold cross-validation using deviance as the evaluation metric. Subsequently, we extracted the non-zero coefficient genes to establish the final feature gene set, excluding the intercept term.

For SVM-RFE analysis, we employed the “e1071,” “kernlab,” and “caret” R packages. A radial basis function kernel was used, and classification performance was evaluated using 10-fold cross-validation with root mean square error (RMSE) as the metric. Feature subset evaluation included specific gene numbers (2, 4, 6, and 8 genes) and continuous sequences (10–40 genes with a step size of 3). The feature gene combination that minimized cross-validation RMSE was selected as the optimal feature set.

RF analysis was conducted using the “randomForest” R package with parameters set as follows: 500 initial decision trees (ntree=500), default mtry parameter (square root of features), and classification mode. The optimal number of trees was determined by minimizing the out-of-bag (OOB) error, followed by model reconstruction. Feature importance was assessed by calculating the mean decrease in Gini index, and genes with importance scores >2 were identified as significant features. The area under the receiver operating characteristic (ROC) curve was visualized using the “pROC” R package (24).

#### 2.1.10 Construction and validation of a nomogram model

A nomogram model was established using the “rms” R package to predict the risk of developing AS, and its predictive power was estimated by using calibration curves and DCA.

#### 2.1.11 Validation of hub Genes

The GSE28829, GES43292 and GSE57691 datasets were used for external validation, and the Wilcoxon test was used to compare the differences in expression between the two groups, with *P*<0.05 considered significant.

#### 2.1.12 Single-cell data analysis

Single-cell sequencing of scRNA-seq data from the GSE159677 dataset was performed using the “Seurat” R package to detect marker genes in each cell cluster (25). Using the “Seurat” R package, initial quality control was performed by excluding cells with fewer than 300 or more than 7500 detected genes, as well as cells with granulocyte gene proportions greater than 15% or hemoglobin gene proportions greater than 1%. Data were normalized using LogNormalize (scale factor=10,000) and the top 2,500 highly variable genes were selected via variance-stabilizing transformation. Dimensionality reduction employed principal component analysis (20 PCs selected by elbow method) followed by cell clustering (Shared Nearest Neighbor algorithm, resolution=0.3) and UMAP visualization (n.neighbors=30, min.dist=0.3). Cell populations were annotated using established marker genes (**Supplementary Table S1**), and cellular proportions across experimental groups were quantified and visualized using stacked bar plots.

#### 2.1.13 Gene-drug interaction

Imported the ASLMRFeGs and Hub genes into the DGIbd database (https://dgidb.org/) to obtain drug candidates and screen for FDA-approved drugs (26).

#### 2.1.14 Molecular docking

The 3D structure of drugs was obtained from the PubChem database (https://pubchem.ncbi.nlm.nih.gov/), and the crystal structure of protein was obtained from the PDB database (https://www.rcsb.org/), and the protein receptor and small molecule ligand were dehydrated and hydrogenated using PyMOL software, and then the molecular docking analysis was performed using AutoDockTools and PyMOL software for visualization. The drugs were molecularly docked to the Hub genes, and the binding energy was calculated; the smaller the binding energy, the better and more stable the binding.

#### 2.1.15 Statistical analysis

All bioinformatics statistical analyses were performed using R software, and *P*<0.05 was considered significant.

### 2.2 Experimental validation

#### 2.2.1 Reagents and instruments

Experimental cells: Human umbilical vein endothelial cells (HUVECs) (Procell, Cat.CL-0675); Mouse RAW 264.7 cells (iCell, Cat.CL-0190).

Reagents: Oxidized low-density lipoprotein (ox-LDL) (Yiyuan Biotechnology, Cat.YB-002-1); Fetal bovine serum (Biosharp, Cat.BL205A); Trypsin-EDTA Solution (Biosharp, Cat.BL512A); RPMI-1640 medium (gibco, Cat.C11875500BT); DMEM medium (Pricella, Cat.PM150210); Penicillin/Streptomycin (Biosharp, Cat.BL505A); Oil red O (ORO) staining Kit (Solarbio, Cat.G1262); Paraformaldehyde, 4% (Solarbio, Cat.P1110); Cell count kit-8 (CCK-8) (Solarbio, Cat.CA1210); ROS detection Kit (BestBio, Cat. BB-47053); Mito-FerroGreen (DOJINDO, Cat.M489); Mounting medium, antifading (with DAPI) (Solarbio, Cat.S2110); Ferrostatin-1 (Fer-1) (APExBIO, Cat.A4371); JC-1 (Beyotime,Lot.C2003S); Serum-free cell freezing medium (Biosharp, Cat.BL203B); RNA extraction solution (Servicebio, Cat.G3013); Phosphate Buffered Saline (Servicebio, Cat.G4202); Chloroform substitute (Servicebio, Cat.G3014); RNA lysate (Servicebio, Cat.G3029); Water Nuclease-Free (Servicebio, Cat.G4700); SweScript All-in-One RT SuperMix for qPCR (One-Step gDNA Remover) (Servicebio, Cat.G3337); 2×Universal Blue SYBR Green qPCR Master Mix (Servicebio, Cat.G3326); isopropanol (Sinopharm, Cat.80109218); Anhydrous ethanol (Sinopharm, Cat.10009218).

Instruments: inverted fluorescence microscope (Nikon, Eclipse Ci); microplate reader (Tecan, Spark 10M); vortex mixer (Servicebio, SMV-3500); sealing instrument (Servicebio, FS-A20); microplate centrifuge (Servicebio, SMP-2); High-speed frozen microcentrifuge (DragonLab, D3024R); fluorescent quantitative PCR instrument (Bio-rad, CFX Connect); PCR instrument (Eastwin, ETC811).

#### 2.2.2 Cell culture

Cells were cultured in RPMI-1640 medium (for HUVECs) or DMEM medium (for RAW 264.7 cells), supplemented with 10% fetal bovine serum and 1% penicillin/streptomycin and incubated at 37°C in humidified 5% CO_2_. Cells were used from passages 3-8.

Cells were divided into three groups as follows: ①the Control group (CTRL): Cells were cultured normally without any treatment; ②the Model group (MOD): Cells were treated with ox-LDL (100 μg/mL) for 24 hours; ③the Fer-1 group (Fer-1): Cells were treated with ox-LDL (100 μg/mL) and Fer-1 (10 μM) for 24 hours.

#### 2.2.3 ORO staining

Cells were fixed in 4% paraformaldehyde for 30 minutes and subsequently rinsed three times. Afterwards, the cells were stained with ORO for 1 hour at room temperature.

#### 2.2.4 Cell activity assay

Cells were cultured in 96-well plates at a density of 2×10^5^ per well. After treatment with different interventions for 24 hours, 10 µL of CCK-8 was added to each well and incubated at 37°C for 1 hour. The absorbance was measured at 450 nm using a microplate reader.

#### 2.2.5 ROS assay

DCFH-DA was diluted at 1:1000, the 12-well plates were removed from incubator, the medium was taken out. Then we washed cells with phosphate buffered saline for 3 times, 1 mL DCFH-DA was added, the cells were incubated at 37 °C for 2 hours, and then the cells were washed with serum-free medium for 3 times.

#### 2.2.6 Fe^2+^ detection

To detect intracellular mitochondrial Fe^2+^, Mito-FerroGreen were used according to the manufacturer’s protocol. The cells were inoculated in 12-well plates and cultured in a 5% CO_2_ incubator at 37°C. After removing the medium, the cells were washed with serum-free medium for 3 times. Mito-Ferrogreen working solution 5μmol was added and cultured 1 hour in 5% CO_2_ incubator at 37°C. After the supernatant was removed, the cells were washed with serum-free medium for 3 times.

#### 2.2.7 JC-1 staining

JC-1 staining were used according to the manufacturer’s protocol. After the 12-well plates were removed from incubator, the culture medium was sucked out, the cells were washed once by phosphate buffered saline, and 0.5 mL cell culture medium and 0.5 mL JC-1 staining working solution were added, and fully mixed. The cells were incubated at 37 °C for 20 minutes in the incubator. After, the supernatant was removed and washed with JC-1 dyeing buffer twice. Add cell culture medium 1 mL.

#### 2.2.8 Quantitative real-time polymerase chain reaction

Total RNA was extracted from cell lines using Trizol total RNA isolation reagent (Invitrogen) according to the manufacturer’s specifications and treated with Turbo DNase (Ambion). cDNA was synthesized from total RNA (0.5 mg) using random hexamers with the TaqMan cDNA Reverse Transcription Kit (Applied Biosystems). Primers were designed using Primer Express v3.0 software, and real-time PCR was performed using SYBR Select Master Mix (Applied Bio-Systems). All reactions were carried out on the 7500 Fast Real-Time PCR System (Applied Biosystem). The average of independent analyses for each gene and sample was calculated using the DD threshold cycle (Ct) method and was normalized to the endogenous reference control gene. The above primers were synthesized by Sangon Biotech (Shanghai) Co., Ltd (https://www.sangon.com/) (**Supplementary Table S2**).

#### 2.2.9 Statistical analysis

Fluorescence microscopy was used for observation and photography. Quantification was performed using Image J software, and results were presented as mean ± standard deviation. Statistical analysis was conducted with GraphPad Prism version 9.0 software. For data that met the criteria of normal distribution and homogeneity of variance, one-way analysis of variance (ANOVA) was used to compare differences among groups, followed by Tuke’s HSD *post hoc* test for pairwise comparisons; for data not meeting these criteria, the Kruskal-Wallis H non-parametric test was employed to compare differences between groups. A *P*<0.05 was considered statistically significant.

## 3 Results

### 3.1 Bioinformatics analysis

#### 3.1.1 Ferroptosis in lipid metabolism pathways alters the immune infiltration microenvironment in AS

**Figure 2:**
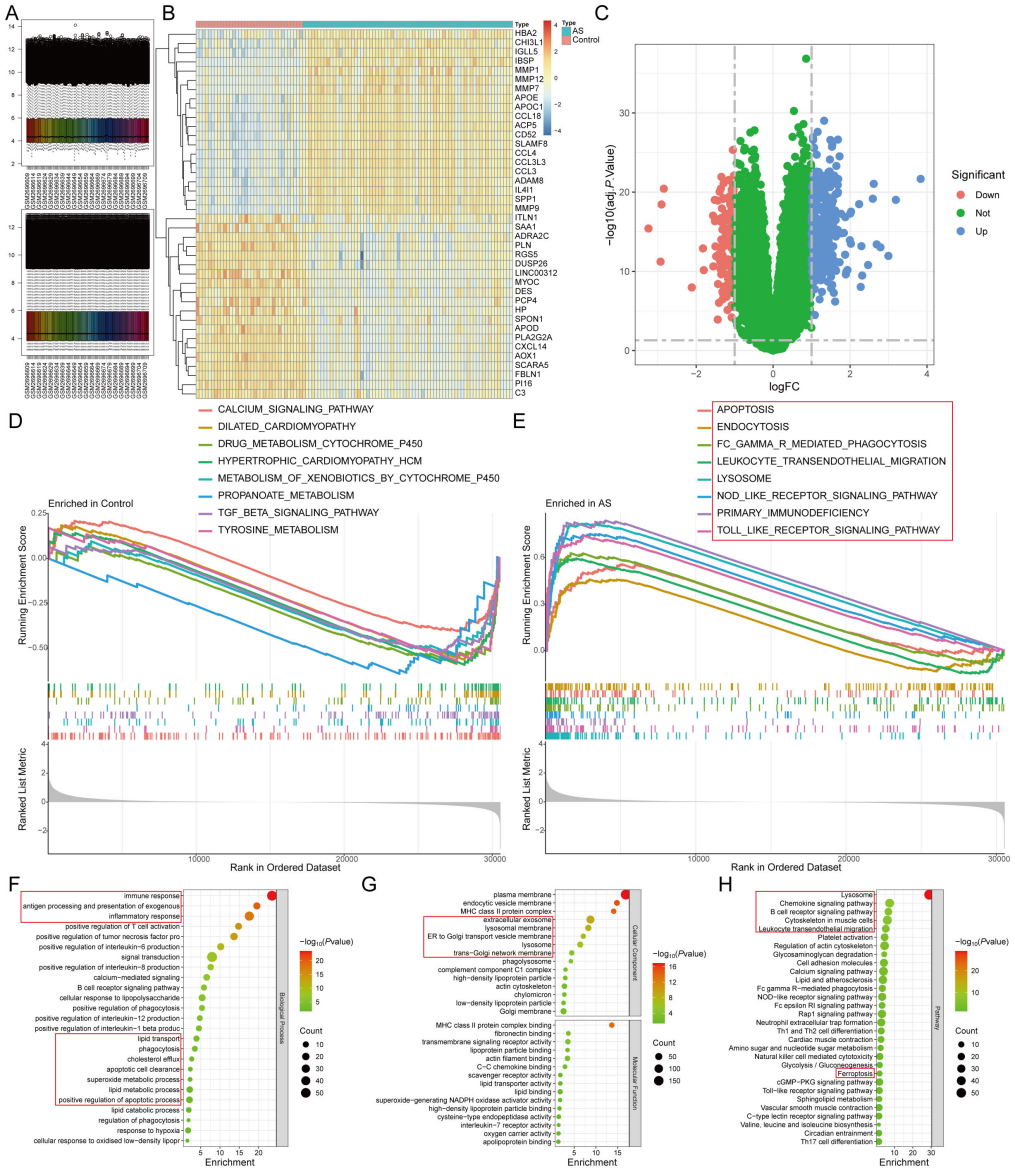
Identification of DEGs of AS by differential expression analysis. **(A)**. The GSE100927 dataset after normalization; **(B, C)**. The **(B)** heatmap and **(C)** volcano plot for DEGs; **(D, E)**. The GSEA for the **(D)** control and **(E)** AS samples. **(F-H)**. The **(F)** biological process, **(G)** cellular component, molecular function, and **(H)** pathway analysis of the DEGs.

Differential gene expression analysis of the AS-related dataset GSE100927 identified 418 DEGs (adj.P<0.05 and |logFC|>1), comprising 295 up-regulated and 123 down-regulated genes (**Figures 2A–C**) (**Supplementary Table S3**). To elucidate the functional implications of these DEGs in AS, we employed the GSEA and KEGG functional enrichment analysis method to uncover differential regulatory pathways between high- and low-expression groups. Our comprehensive analysis disclosed that the pathogenesis of AS chiefly encompasses organelle dysfunction (lysosomes, exosomes, endoplasmic reticulum, Golgi apparatus), cardiomyopathies (dilated cardiomyopathy, hypertrophic cardiomyopathy), metabolic dysregulation (tyrosine and propionic acid metabolism, valine, leucine, and isoleucine biosynthesis, glycosaminoglycan degradation), lipid metabolism disorder (lipids and AS, lipoprotein particle binding, cholesterol efflux, lipolytic metabolic processes), cell death mechanisms (ferroptosis, apoptosis), inflammatory processes (neutrophil extracellular trap formation, positive regulation of tumor necrosis factor, IL-6, IL-8, IL-12, and IL-1β production), and immune dysregulation (primary immunodeficiency, natural killer cell-mediated cytotoxicity, B-cell receptor signaling pathways). These pathways are closely associated with various organelles, such as lysosomes, endoplasmic reticulum (ER), and Golgi apparatus (**Figures 2D–H**).

**Figure 3:**
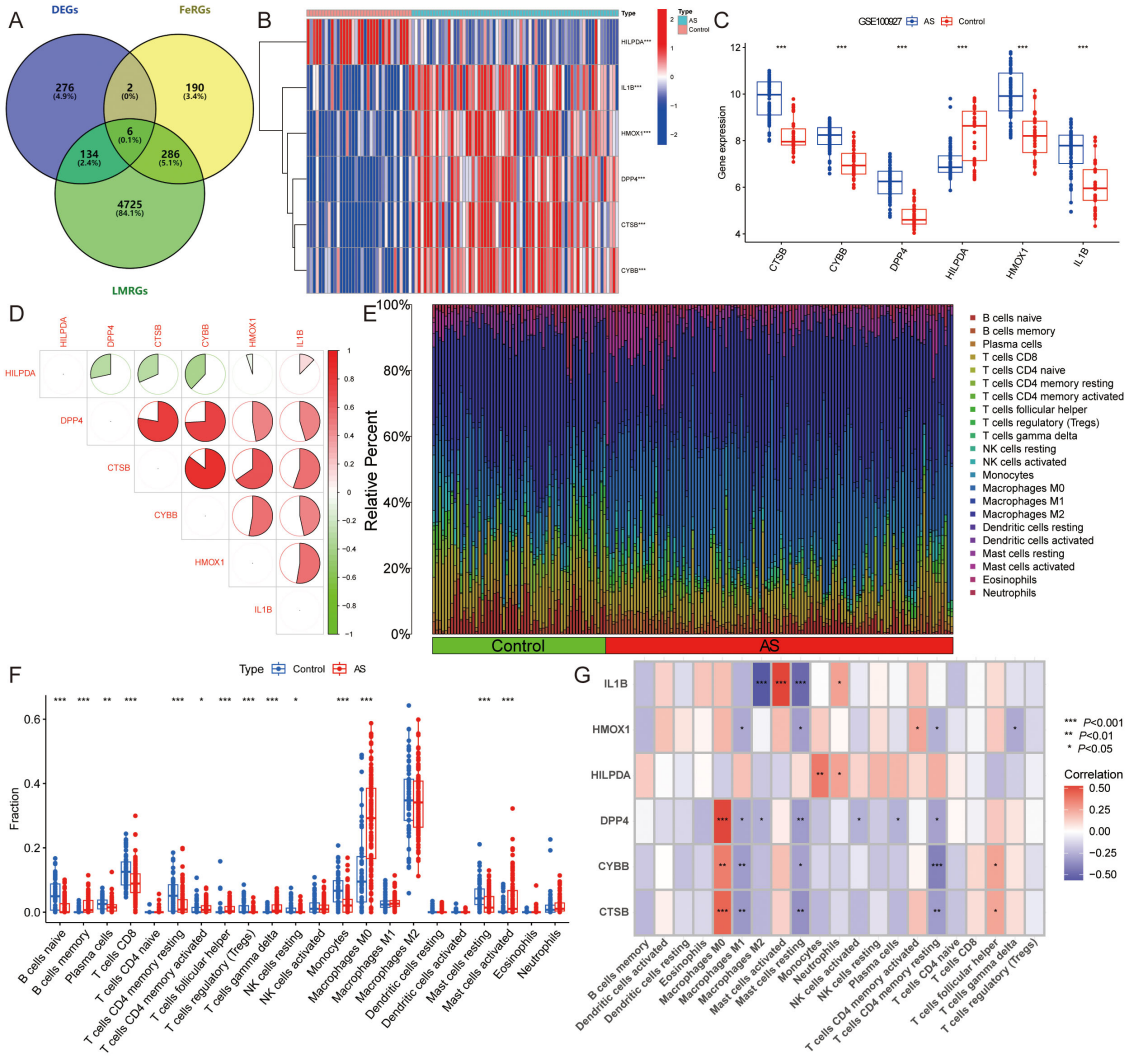
Identification and immune infiltration analysis of AS lipid metabolism-related ferroptosis genes (ASLMRFeGs). **(A)**. Venn diagram showing six ASLMRFeGs; **(B, C)**. The **(B)** heatmap and **(C)** volcano of expression levels of the ASLMRFeGs between the AS and control groups; **(D)**. Correlation analysis between ASLMRFeGs; **(E)**. The degree of immune cell infiltration for each sample; **(F)**. Boxplot showing differences in immune infiltration between AS and control groups; **(G)**. Correlation analysis of ASLMRFeGs and immune cells. ^*^ *P*<0.05, ^**^ *P*<0.001, ^***^ *P*<0.0001.

From the FerrDb database, we retrieved 484 FeRGs (**Supplementary Table S4**), while 5151 LMRGs were obtained from public databases and literature queries (**Supplementary Table S5**). The intersection of these datasets with DEGs yielded six ASLMRFeGs (CTSB, CYBB, DPP4, HILPDA, HMOX1, IL1B) (**Figure 3A**). Among these, CTSB (logFC=1.530), CYBB (logFC=1.116), DPP4 (logFC=1.430), HMOX1 (logFC=1.839), and IL1B (logFC=1.447) exhibited higher expression in AS, whereas HILPDA (logFC=-1.172) displayed lower expression (**Figures 3B, C**). In addition, CTSB, CYBB, DPP4, HILPDA, and IL1B are ferroptosis driver genes, whereas HMOX1 is a bidirectional regulator of ferroptosis (driver and suppressor). To delve deeper into the roles of ASLMRFeGs in AS progression, we conducted correlation analyses. The results illuminated robust synergistic effects among CTSB, CYBB, DPP4, HMOX1, and IL1B, whereas HILPDA exhibited an antagonistic relationship with CTSB, CYBB, DPP4, and HMOX1 (**Figure 3D**).

Expanding our investigation into the immune microenvironment of AS, we analyzed specific immune cell types infiltrating AS tissues using the CIBERSORT method (**Figure 3E**). Our findings revealed significantly elevated levels of memory B cells, activated memory CD4 T cells, follicular helper T cells, γ-δ T cells, M0 macrophages, and activated mast cells in AS. Conversely, significantly lower levels of naive B cells, plasma cells, CD8 T cells, resting memory CD4 T cells, regulatory T cells, resting NK cells, monocytes, and resting mast cells were observed in AS (**Figure 3F**). Furthermore, correlation analysis unveiled significant positive associations of CTSB, CYBB, DPP4, and HMOX1 with M0 macrophages, along with significant negative correlations with M1 macrophages, resting mast cells, and resting memory CD4+ T cells. IL1B showed a negative association with M2 macrophages and resting mast cells but a positive link with activated mast cells (**Figure 3G**). These findings suggest that ASLMRFeGs contribute to the regulation of the immune microenvironment in AS by influencing specific immune cell infiltration and activation states.

#### 3.1.2 Identification of lipid metabolism pathway ferroptosis-related molecular clusters in AS

**Figure 4:**
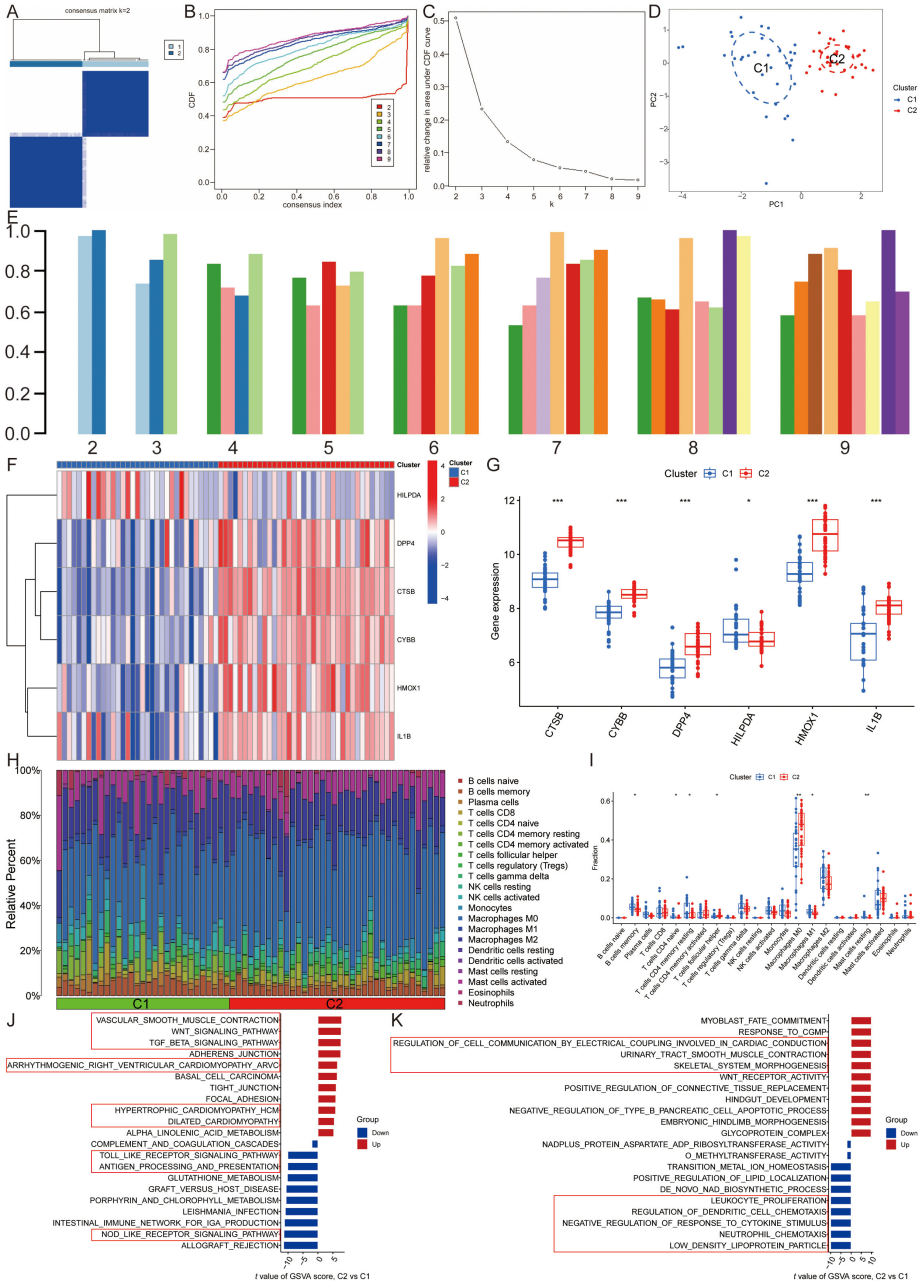
Identification of lipid metabolism pathway ferroptosis-related molecular clusters in AS. **(A)**. Heatmap of the consensus clustering matrix for k=2; **(B-D)**. The **(B)** cumulative distribution function (CDF) curves, **(C)** CDF delta area curves, **(D)** PCA, and **(E)** the score of consensus clustering; **(F, G)**. The **(F)** heatmap and **(G)** boxplot of expression levels of the ASLMRFeGs between two clusters; **(H)**. The degree of immune cell infiltration in each AS sample; **(I)**. Comparison of immune cell infiltration between two clusters; **(J, K)** The GSVA analysis of the C1 and C2 clusters demonstrates differences in **(J)** pathways and **(K)** biological processes. ^*^ *P*<0.05, ^**^ *P*<0.001, ^***^ *P*<0.0001.

To classify the 69 AS samples based on the expression profiles of the six ASLMRFeGs, a consensus clustering algorithm was employed, resulting in the identification of two distinct and stable groups: the C1 cluster (*n*=33) and the C2 cluster (*n*=36) (**Figures 4A–E**). Further analysis of the expression differences of the six ASLMRFeGs between the C1 and C2 clusters revealed that CTSB, CYBB, DPP4, HMOX1, and IL1B were highly expressed in the C2 cluster, while HILPDA was highly expressed in the C1 cluster (**Figures 4F, G**).

Immune infiltration analyses of the two clusters demonstrated that the C1 cluster had a significantly higher abundance of memory B cells, naive CD4 T cells, resting memory CD4 T cells, M1 macrophages, and resting mast cells. In contrast, the C2 cluster exhibited significantly higher levels of follicular helper T cells and M0 macrophages (**Figures 4H, I**).

GSVA was then utilized to investigate potential biological and functional differences between the two clusters. The results indicated that the C2 cluster was primarily enriched in pathways related to inflammation and immune response, including leukocyte proliferation, regulation of dendritic cell chemotaxis, neutrophil chemotaxis, antigen processing and presentation, the Toll-like receptor signaling pathway, and the NOD-like receptor signaling pathway. Conversely, the C1 cluster was mainly associated with pathways affecting the structure and function of the cardiovascular system and cell signaling, such as glycoprotein complex, regulation of cell communication by electrical coupling involved in cardiac conduction, dilated cardiomyopathy, hypertrophic cardiomyopathy, arrhythmogenic right ventricular cardiomyopathy, vascular smooth muscle contraction, the TGF-beta signaling pathway, and the Wnt signaling pathway (**Figures 4J, K**). These findings suggest that, based on the expression of ASLMRFeGs, AS samples can be divided into two subgroups with significantly different biological functions, particularly in terms of inflammation and immune response.

#### 3.1.3 Identification and functional enrichment analysis of candidate module hub genes

**Figure 5:**
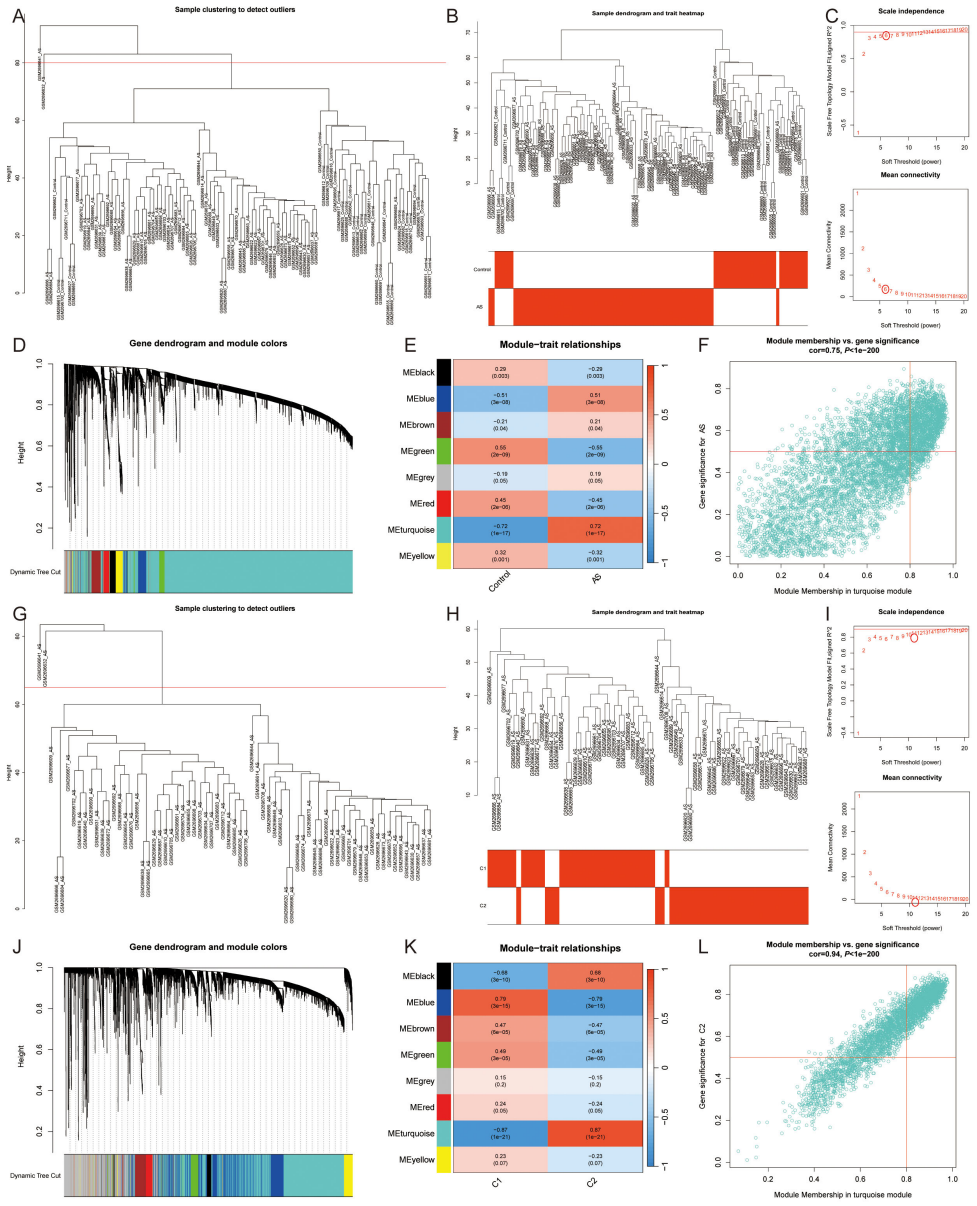
Co-expression network of DEGs and two lipid metabolism pathway ferroptosis-related molecular clusters. **(A, B, G, H)**. **(A, B)** All samples and **(G, H)** AS samples clustering plot after removing outlier samples; **(C, I)**. The selection of soft threshold power; **(D, J)**. Dendrogram of all genes clustered based on the measurement of dissimilarity (1-TOM); **(E, K)**. Correlation analysis between module eigengenes and clinical status; **(F, L)**. Scatter plot between module membership in the turquoise module and the gene significance for **(F)** AS or **(L)** C1 Cluster.

By utilizing WGCNA, co-expression networks and modules were constructed for both control and AS samples (**Figures 5A, B**). Upon setting the soft threshold to 6, 8 distinct modules with different colors were identified, among which the “turquoise” module exhibited the strongest positive correlation with AS (*r*=0.72) (**Figures 5C-E**). This particular module comprised 6069 module genes and 1425 module Hub genes (**Figure 5F**). Moreover, WGCNA analysis revealed module Hub genes that were associated with lipid metabolism pathway ferroptosis in AS samples (**Figures 5G, H**). When the soft threshold was set to 11, a total of 8 modules with distinct colors were detected. Among them, the “black,” “blue,” “brown,” “green,” and “turquoise” modules are significantly correlated with the C2 cluster, and once again, the “turquoise” module showcased the highest positive correlation with the C2 cluster (*r*=0.87) (**Figures 5I–K**). In order to screen the most relevant molecular modules for lipid metabolism and ferroptosis, we enriched the genes in the different modules and showed that the “turquoise” module was closely related to inflammatory response, immune response, lipid metabolism, and ferroptosis; the “black” module was related to cell cycle; the “blue” module was related to muscle contraction, regulation of actin cytoskeleton, and cell signaling; and the “green” module was related to cell adhesion and angiogenesis, while the “brown” module was not significantly enriched. Therefore, the “turquoise” module, which includes 3284 module genes and 1515 module Hub genes, was used for the study (**Figure 5L**) (**Supplementary Table S6**).

**Figure 6:**
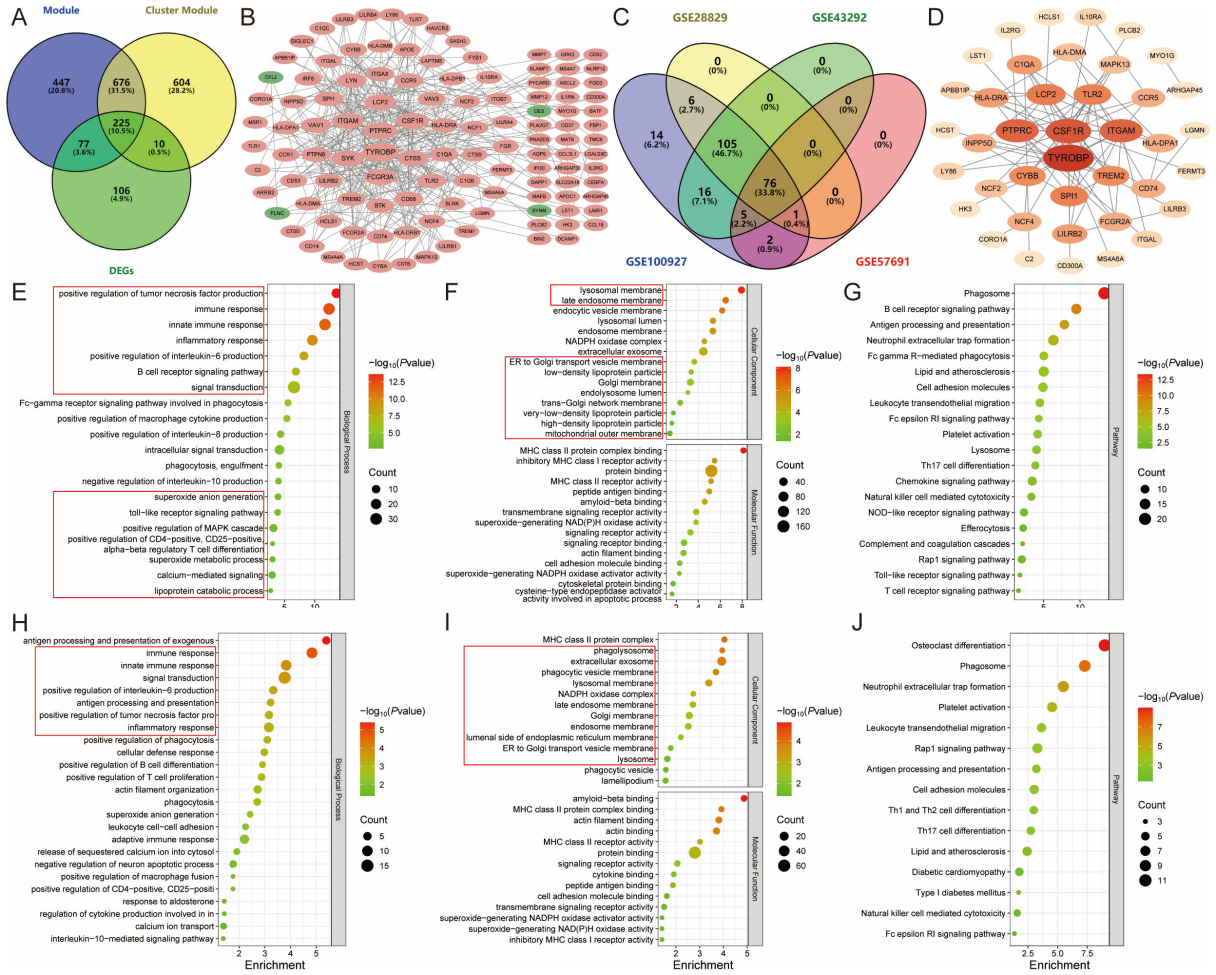
Identification and functional enrichment analysis of module Hub genes. **(A)**. Venn diagram showing the 225 candidate module Hub genes; **(B)**. PPI network of candidate module Hub genes, with pink representing upregulated genes and green representing downregulated genes; **(C)**. External validation screening module Hub genes; **(D)**. PPI network of module Hub genes, with colors darkening according to degree centrality values; **(E-G)**. The **(E)** biological process, **(F)** cellular component, molecular function, and **(G)** pathway analysis of candidate module Hub genes; **(H-J)**. The **(H)** biological process, **(I)** cellular component, molecular function, and **(J)** pathway analysis of module Hub genes.

The intersection of module genes from the two “turquoise” modules and DEGs resulted in 225 candidate module Hub genes (**Figure 6A**), and constructed a PPI network with 118 nodes and 359 edges (**Figure 6B**). The analysis of these genes using gene ontology and KEGG revealed their involvement in various biological functions and pathways. The gene ontology analysis encompassed three main categories: biological process, cellular component, and molecular function. Among the 225 candidate module Hub genes, 189 were linked to biological processes, 55 to cellular components, and 41 to molecular functions, while the KEGG enrichment analysis identified 52 signaling pathways (*P*<0.05). The biological process analysis indicated the participation of these genes in several processes, including inflammatory response, immune response, signal transduction, the positive regulation of tumor necrosis factor, macrophage cytokine, IL-6, IL-8, and IL-10 production, and lipoprotein catabolic process. The cellular component analysis highlighted the role of various cellular membranes and organelles, especially the lysosomal membrane, late endosome membrane, endocytic vesicle membrane, endosome membrane, ER-to-Golgi transport vesicle membrane, Golgi membrane, and mitochondrial outer membrane. Furthermore, the molecular function analysis emphasized their involvement in MHC class II protein complex binding, transmembrane signaling receptor activity, superoxide-generating NAD(P)H oxidase activity, signaling receptor activity, actin filament binding, and cysteine-type endopeptidase activator activity involved in apoptotic processes (**Figures 6E, F**). The pathway analysis revealed enrichment in various pathways, including the B cell receptor signaling pathway, the Fc epsilon RI signaling pathway, the chemokine signaling pathway, the NOD-like receptor signaling pathway, the Toll-like receptor signaling pathway, the T cell receptor signaling pathway, the Rap1 signaling pathway, Th17 cell differentiation, natural killer cell-mediated cytotoxicity, complement and coagulation cascades, lysosome, phagosome, efferocytosis, neutrophil extracellular trap formation, Fc gamma R-mediated phagocytosis, leukocyte transendothelial migration, and lipid and AS (**Figure 6G**). The above results show that the pathogenesis of ferroptosis in the AS lipid metabolism pathway involves abnormalities in several biological processes, including cellular communication, immune response, and metabolic response.

#### 3.1.4 Screening and functional enrichment analysis of module hub genes

To further screen the candidate Hub genes, 225 candidate module Hub genes were externally validated by single-gene differential analysis in the GSE43292, GSE28829, and GSE57691 datasets. This validation revealed that 76 module Hub genes were significantly differentially expressed across all four datasets (**Figures 6C, D**) (**Supplementary Table S7**). These 76 genes were then imported into the DAVID database for gene ontology and KEGG pathway analysis. The results showed that 61 genes were correlated with biological processes, 36 with cellular components, and 14 with molecular functions. Additionally, KEGG enrichment analysis identified 38 signaling pathways (*P*<0.05).

The biological process analysis indicated involvement in several processes, including immune response, inflammatory response, signal transduction, positive regulation of phagocytosis, actin filament organization, positive regulation of tumor necrosis factor and interleukin-6 production, superoxide anion generation, leukocyte cell-cell adhesion, negative regulation of neuron apoptotic process, positive regulation of macrophage fusion, and interleukin-10-mediated signaling pathway. The cellular component analysis identified the phagolysosome, late endosome membrane, Golgi membrane, endosome membrane, ER-to-Golgi transport vesicle membrane, lysosome, phagocytic vesicle, and lamellipodium. The molecular function analysis highlighted amyloid-beta binding, MHC class II protein complex binding, actin binding, signaling receptor activity, cytokine binding, and superoxide-generating NADPH oxidase activator activity (**Figures 6H, I**).

Pathway analysis indicated enrichment in the Rap1 signaling pathway, the Fc epsilon RI signaling pathway, phagosome, neutrophil extracellular trap formation, leukocyte transendothelial migration, cell adhesion molecules, Th1 and Th2 cell differentiation, Th17 cell differentiation, lipid and AS, and natural killer cell-mediated cytotoxicity. These findings suggest that AS may modulate lipid metabolism and ferroptosis by regulating inflammation and immune responses (**Figure 6J**).

#### 3.1.5 Machine learning screening of candidate hub genes

**Figure 7:**
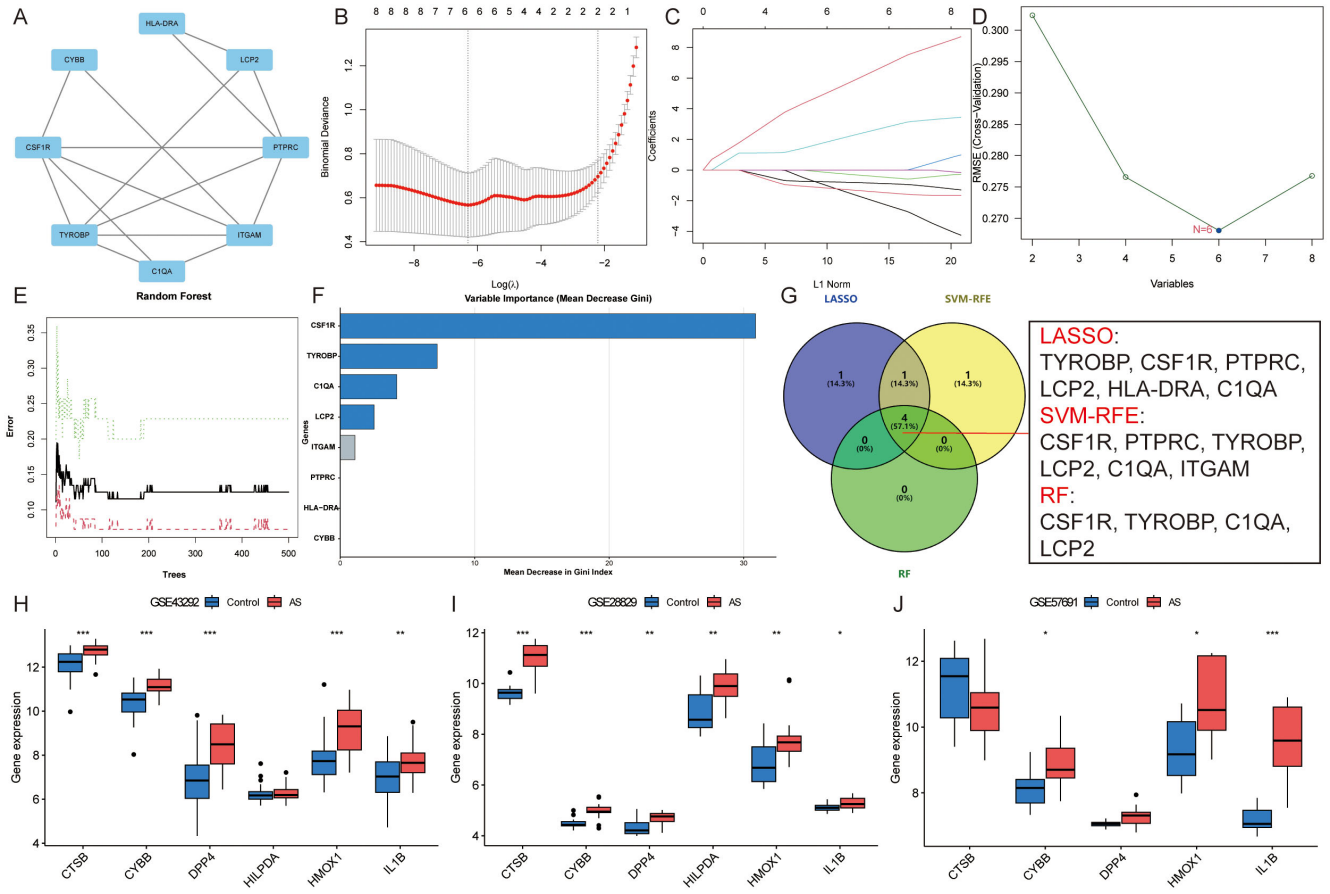
Identification of candidate Hub genes. **(A)**. PPI network of candidate Hub genes; **(B, C)**. LASSO regression identified six key genes; **(D)**. SVM-RFE identified six featured genes; **(E, F)**. RF obtained four important genes; **(G)**. Venn diagram showing the four candidate Hub genes; **(H-J)**. External verification of ASLMRFeGs in the **(H)** GSE43292, **(I)** GSE28829, and **(J)** GSE57691 datasets. Specifically, * indicates P<0.05, ** indicates P<0.001, and *** denotes P<0.0001.

Subsequently, these 76 module Hub genes were imported into the STRING database to construct a PPI network with a “score” ≥ 0.7, which had 40 nodes and 79 edges (**Figure 6D**). 8 candidate Hub genes (TYROBP, CSF1R, PTPRC, ITGAM, LCP2, CYBB, HLA-DRA, and C1QA) with degree centrality, betweenness centrality, and closeness centrality all greater than the mean were filtered out (**Supplementary Table S8**), and the constructed PPI network of candidate Hub genes includes 8 nodes and 15 edges (**Figure 7A**). To further refine the selection of characterized genes, 3 types of machine learning algorithms were employed: LASSO regression, SVM-RFE, and RF. LASSO regression identified 6 key genes (TYROBP, CSF1R, PTPRC, LCP2, HLA-DRA, and C1QA) (**Figures 7B, C**), SVM-RFE identified 6 featured genes (CSF1R, PTPRC, TYROBP, LCP2, C1QA, and ITGAM) (**Figure 7D**), and RF obtained 4 important genes (CSF1R, TYROBP, C1QA, and LCP2) (**Figures 7E, F**) (**Supplementary Table S9**). Ultimately, an intersection of the genes identified by these methods yielded 4 characterized genes: TYROBP, CSF1R, LCP2, and C1QA, which were all up-regulated in AS (**Figure 7G**).

Similarly, external validation of single-gene differential analysis of six ASLMRFeGs in the GSE43292, GSE28829, and GSE57691 datasets showed that CYBB, HMOX1, and IL1B were significantly differentially expressed in these datasets and up-regulated in AS. Therefore, we considered these three ASLMRFeGs and four characterized genes as candidate Hub genes, namely, CYBB, HMOX1, IL1B, CSF1R, TYROBP, C1QA, and LCP2 (**Figures 7H–J**).

### 3.2 Experimental validation

#### 3.2.1 Inhibition of ferroptosis attenuates ox-LDL-induced lipid accumulation and increases cell activity in HUVECs and RAW 264.7 cells

**Figure 8:**
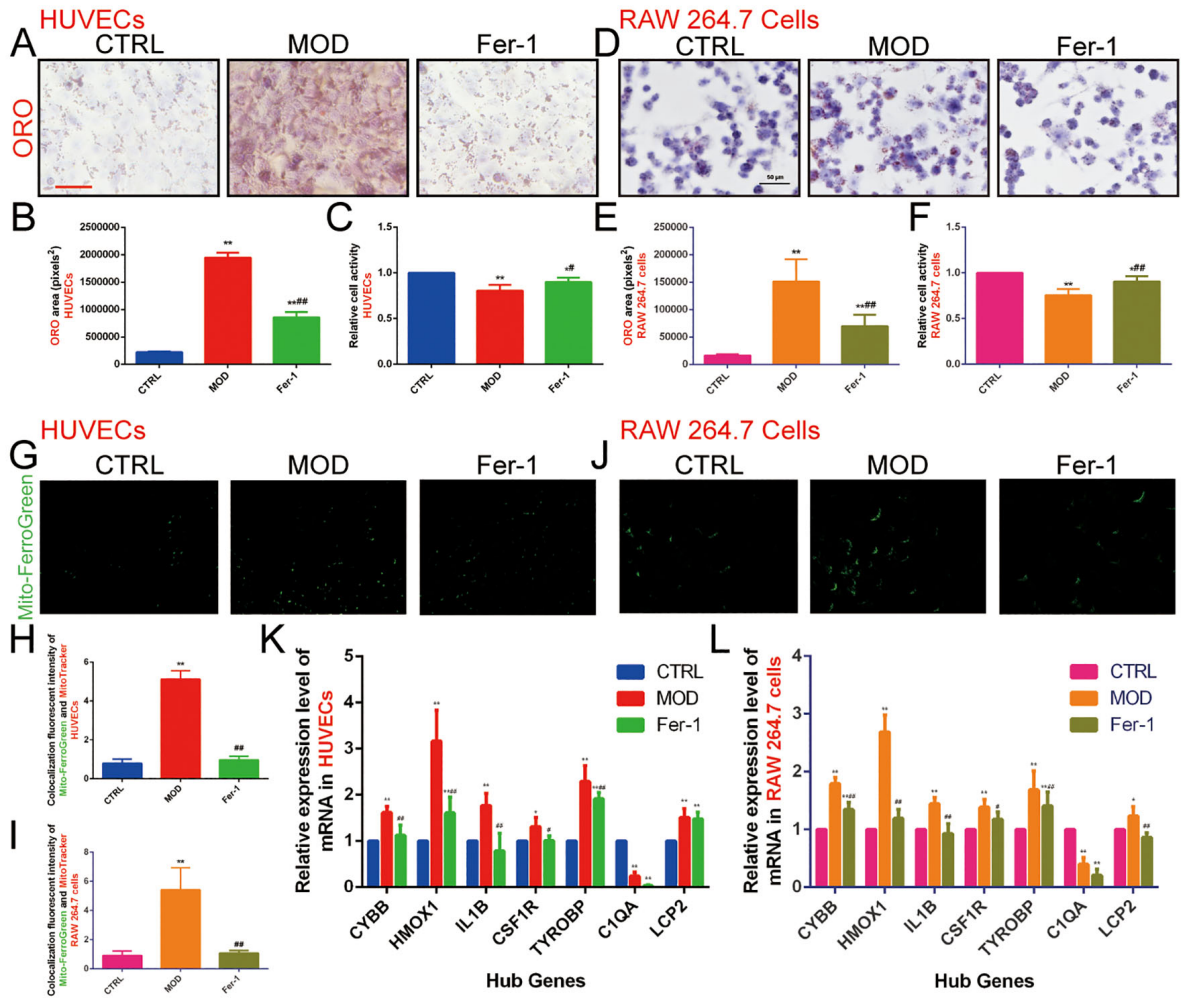
Effect of ox-LDL on ferroptosis in HUVECs and RAW 264.7 cells. **(A, B)**. ORO staining of HUVECs (n=9, bar=500 μm); **(C)**. CCK-8 to detect the relative activity in HUVECs (n=9); **(D, E)**. ORO staining of RAW 264.7 cells (n=9, bar=50 μm); **(F)**. CCK-8 to detect the relative activity in RAW 264.7 cells (n=9); **(G-I)**. Mito-FerroGreen to detect mitochondrial Fe^2+^ content in **(G, H)** HUVECs and **(I, J)** RAW 264.7 cells (n=5, bar=500μm); **(K, L)**. qRT-PCR to detect the relative expression levels of ASLMRFeGs and Hub genes in **(K)** HUVECs and **(L)** RAW 264.7 cells (n=9). Results are expressed as the mean ± S.D. ^*^ *P*<0.05 vs. the CTRL group, ^**^ *P*<0.01 vs. the CTRL group, ^#^ *P*<0.05 vs. the MOD group, ^##^ *P*<0.01 vs. the MOD group.

We validated this using the RAW 264.7 and HUVECs dual cell lines, one belonging to endothelial cells and one to macrophages. Firstly, we needed to confirm that ox-LDL-induced HUVECs and RAW 264.7 cells clearly caused abnormal lipid metabolism, so ORO staining was used to detect intracellular lipid accumulation, and CCK-8 was used to detect cell activity. The results showed that there was only a small amount of ORO staining observed in normal HUVECs and RAW 264.7 cells, and extensive ORO staining was visible in the cytoplasm of ox-LDL-treated HUVECs and RAW 264.7 cells in the MOD group, with increased cell volume and rounded or irregular morphology. The relative area of ORO was significantly increased (*P*<0.01) and cell activity was significantly decreased (*P*<0.01) in the MOD group compared with the CTRL group, suggesting that ox-LDL-induced HUVECs and RAW 264.7 cells resulted in an intracellular abnormality of lipid metabolism, which led to significant lipid accumulation and decreased cell activity in the cells (**Figures 8A–F**).

To verify whether abnormal lipid metabolism could promote ferroptosis, we used Mito-FerroGreen to detect mitochondrial Fe^2+^ content. The results showed that, compared with the CTRL group, the MOD group significantly elevated mitochondrial Fe^2+^ content (*P*<0.01), suggesting that ox-LDL induces ferroptosis in HUVECs and RAW 264.7 cells and that abnormalities in lipid metabolism promote ferroptosis (**Figures 8G–J**). Then, we used Fer-1 (an inhibitor of ferroptosis) for subsequent experiments, and the results showed that the Fer-1 group significantly alleviated intracellular lipid accumulation (*P*<0.01), elevated cell activity (*P*<0.05), and reduced mitochondrial Fe^2+^ content (*P*<0.01) compared with the MOD group, suggesting that inhibition of ferroptosis improves lipid metabolism and cell activity to a certain extent (**Figures 8A–F**).

In order to predict the potential mechanism of lipid metabolism in AS and ferroptosis, we used qRT-PCR to examine the expression levels of ASLMRFeGs and Hub genes. The results showed that ox-LDL treatment (MOD group) significantly up-regulated the expression of CYBB, HMOX1, IL1B, CSF1R, TYROBP, and LCP2 genes, while down-regulating C1QA gene compared with the CTRL group in both HUVECs and RAW 264.7 cells. Treatment with the ferroptosis inhibitor Ferrostatin-1 (Fer-1 group) significantly attenuated this ox-LDL-induced upregulation of CYBB, HMOX1, IL1B, CSF1R, and TYROBP genes compared with the MOD group in both cell lines. Additionally, Fer-1 attenuated LCP2 upregulation specifically in RAW 264.7 cells (**Figures 8K, L**).

#### 3.2.2 Inhibition of ferroptosis improves ox-LDL-induced mitochondrial and lysosomal dysfunction and ER stress overactivation in HUVECs and RAW 264.7 cells

The previous bioinformatics analysis results indicated that the mechanisms by which lipid metabolism regulates AS and ferroptosis are closely related to various organelles, particularly mitochondria, lysosomes, the ER, and the Golgi apparatus. Therefore, we investigated the role of mitochondria using three different methods: the JC-1 fluorescent dye to measure the relative fluorescence intensity ratio (red/green fluorescence) of mitochondrial membrane potential, with a higher ratio indicating better mitochondrial function; DCFH-DA fluorescent staining to assess the content of ROS; and qRT-PCR to detect the relative expression level of mtDNA-ND1 in mitochondria to determine mitochondrial function.

**Figure 9:**
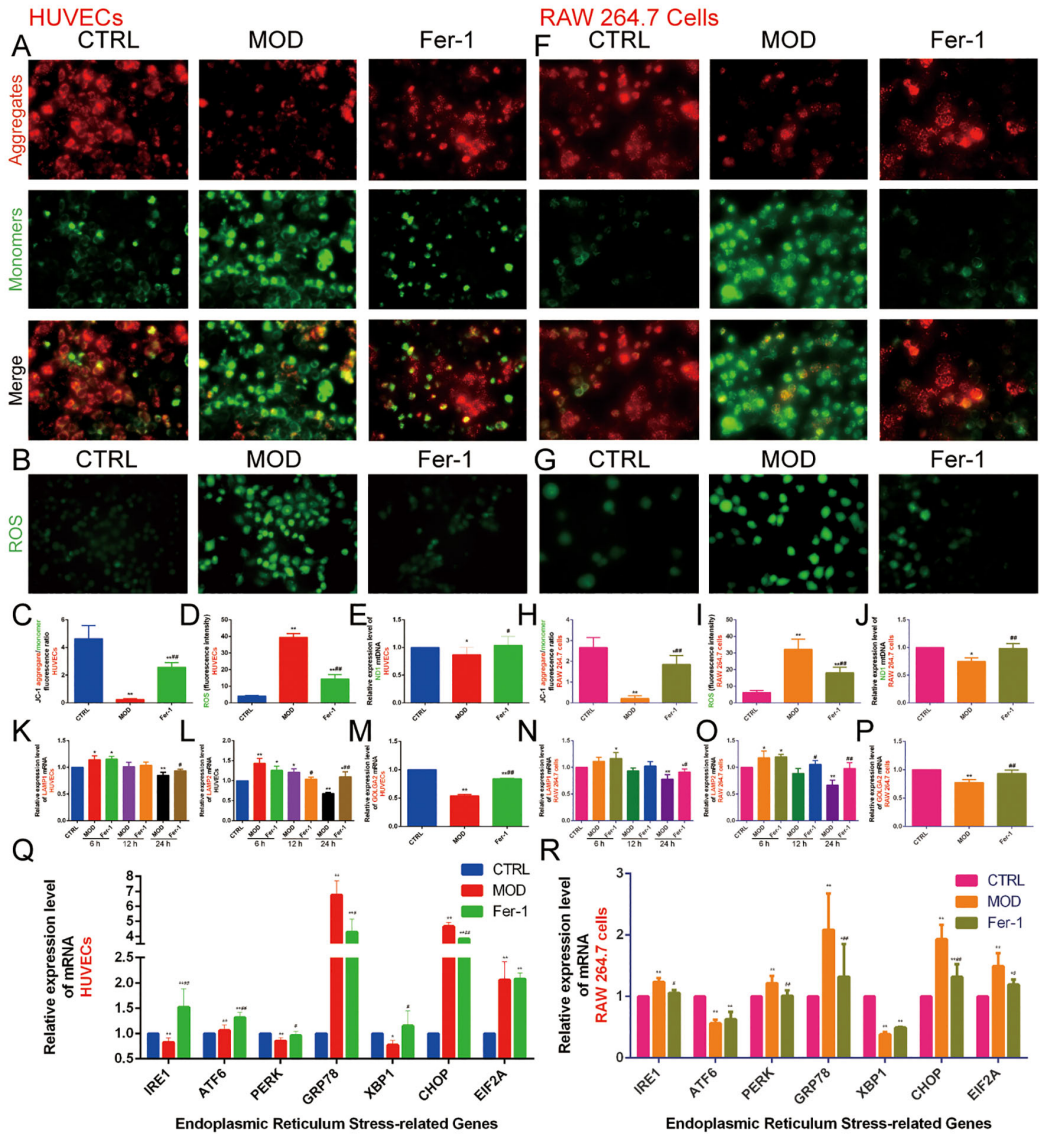
Effect of ox-LDL on mitochondria, lysosomes, Golgi apparatus, and endoplasmic reticulum in HUVECs and RAW 264.7 cells. **(A, C)**. JC-1 staining to detect mitochondrial membrane potential in HUVECs (n=9, bar=500μm); **(B, D)**. DCFH-DA fluorescent staining to detect ROS content in HUVECs (n=9, bar=500μm); **(E)**. qRT-PCR to detect the relative expression levels of mitochondrial mtDNA ND1 in HUVECs (n=9); **(F, H)**. JC-1 staining to detect mitochondrial membrane potential in RAW 264.7 cells (n=9, bar=500μm); **(G, I)**. DCFH-DA fluorescent staining to detect ROS content in RAW 264.7 cells (n=9, bar=500μm); **(J)**. qRT-PCR to detect the relative expression levels of mitochondrial mtDNA ND1 in RAW 264.7 cells (n=9); **(K-M)**. qRT-PCR to detect the relative expression levels of **(K)** LAMP1, **(L)** LAMP2, and **(M)** GOLGA2 in HUVECs (n=9); **(N-P)**. qRT-PCR to detect the relative expression levels of **(N)** LAMP1, **(O)** LAMP2, and **(P)** GOLGA2 in RAW 264.7 cells (n=9); **(Q-R)**. qRT-PCR to detect the relative expression levels of IRE1, ATF6, PERK, GRP78, XBP1, CHOP, and EIF1A in **(Q)** HUVECs and **(R)** RAW 264.7 cells (n=9). Results are expressed as the mean ± S.D. ^*^ *P*<0.05 vs. the CTRL group, ^**^ *P*<0.01 vs. the CTRL group, ^#^ *P*<0.05 vs. the MOD group, ^##^ *P*<0.01 vs. the MOD group.

The results showed that the fluorescence intensity ratio of mitochondrial membrane potential was significantly weaker, the fluorescence intensity of ROS was significantly stronger, and the relative expression level of ND1 in mitochondria was decreased in the MOD group compared with the CTRL group. This suggests that ox-LDL-induced mitochondrial dysfunction in HUVECs (**Figures 9A–E**) and RAW 264.7 cells (**Figures 9F–J**) promote ROS production. However, Fer-1 antagonized the mitochondrial-damaging effects induced by ox-LDL, indicating that inhibition of ferroptosis improved mitochondrial dysfunction induced by abnormalities in lipid metabolism.

Next, we found that 6 hours of ox-LDL intervention upregulated the relative expression of LAMP1 and LAMP2 genes in HUVECs and the LAMP2 gene in RAW 264.7 cells. At 12 hours, ox-LDL intervention continued to upregulate LAMP2 gene expression in HUVECs. However, after 24 hours of ox-LDL intervention, both LAMP1 and LAMP2 gene expression were downregulated in both HUVECs (**Figures 9K, L**) and RAW 264.7 cells (**Figures 9N, O**). In contrast, Fer-1 treatment increased the expression of both LAMP1 and LAMP2 genes. These findings suggest that abnormal lipid metabolism may induce lysosomal activation in the early stages, followed by lysosomal damage in later stages. Inhibition of ferroptosis may help mitigate lysosomal damage.

GOLGA2 is a classical marker of the Golgi apparatus. We found that the MOD group downregulated GOLGA2 gene expression compared with the CTRL group, whereas Fer-1 upregulated GOLGA2 gene expression compared with the MOD group (**Figures 9M, P**).

The ER is an important organelle for lipid and protein synthesis. Under various stimuli, overloaded protein synthesis exceeds the storage capacity of the ER, promoting ERS. GRP78 and CHOP are commonly used as markers of ERS. We also detected genes of the three pathways of ERS (IRE1, ATF6, PERK, XBP1, and EIF2A). The results indicated that in HUVECs, the MOD group significantly increased the expression of ATF6, GRP78, CHOP, and EIF2A genes while decreasing the expression of IRE1, PERK, and XBP1 genes compared to the CTRL group. In contrast, the Fer-1 group upregulated IRE1, ATF6, PERK, and XBP1 genes and downregulated GRP78 and CHOP gene expression compared to the MOD group. In RAW 264.7 cells, the MOD group upregulated IRE1, PERK, GRP78, CHOP, and EIF2A genes while downregulating ATF6 and XBP1 genes compared to the CTRL group. In contrast, the Fer-1 group downregulated IRE1, PERK, GRP78, CHOP, and EIF2A genes compared to the MOD group (**Figures 9Q, R**). This suggests that abnormal lipid metabolism significantly activates ERS, and inhibition of ferroptosis suppresses ERS.

### 3.3 Construction and evaluation of nomogram

**Figure 10:**
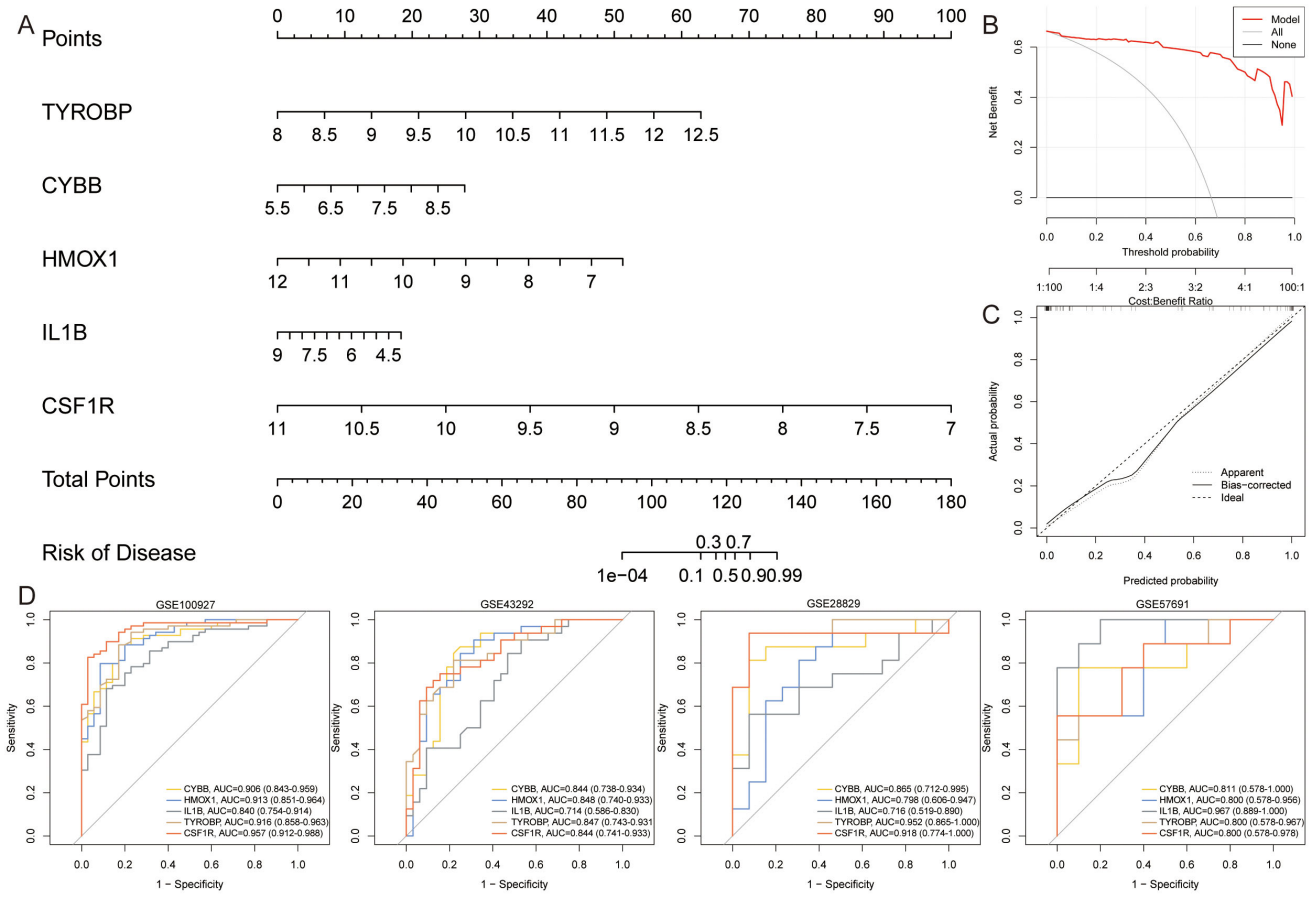
Construction and assessment of nomogram. **(A)**. A nomogram was constructed based on TYROBP, CYBB, HMOX1, IL1B, and CSF1R to predict the risk of developing AS; **(B, C)**. Construction of **(B)** calibration curve and **(C)** DCA for assessing the predictive efficiency of the nomogram; **(D)**. ROC curves for Hub genes in the external validation set.

We constructed a nomogram to assess the role of ASLMRFeGs and Hub genes in predicting the risk of AS development (**Figure 10A**). Previous bioinformatics analyses indicated that TYROBP, CSF1R, LCP2, C1QA, CYBB, HMOX1, and IL1B were upregulated in AS samples. However, our *in vitro* experiments revealed that C1QA was downregulated in the AS model group, and that inhibition of ferroptosis did not improve the expression of C1QA and LCP2. Therefore, we chose TYROBP, CSF1R, CYBB, HMOX1, and IL1B to construct a nomogram to predict the risk of AS. The predictive efficiency of the nomogram was then evaluated using calibration curves and DCA (**Figures 10B, C**). External validation was performed using the GSE28829, GSE43292, and GSE57691 datasets, and the ROC curves demonstrated that these gene-based prediction models performed well (all AUC>0.70), effectively distinguishing normal and AS samples (**Figure 10D**).

### 3.4 Single-cell data analysis

**Figure 11:**
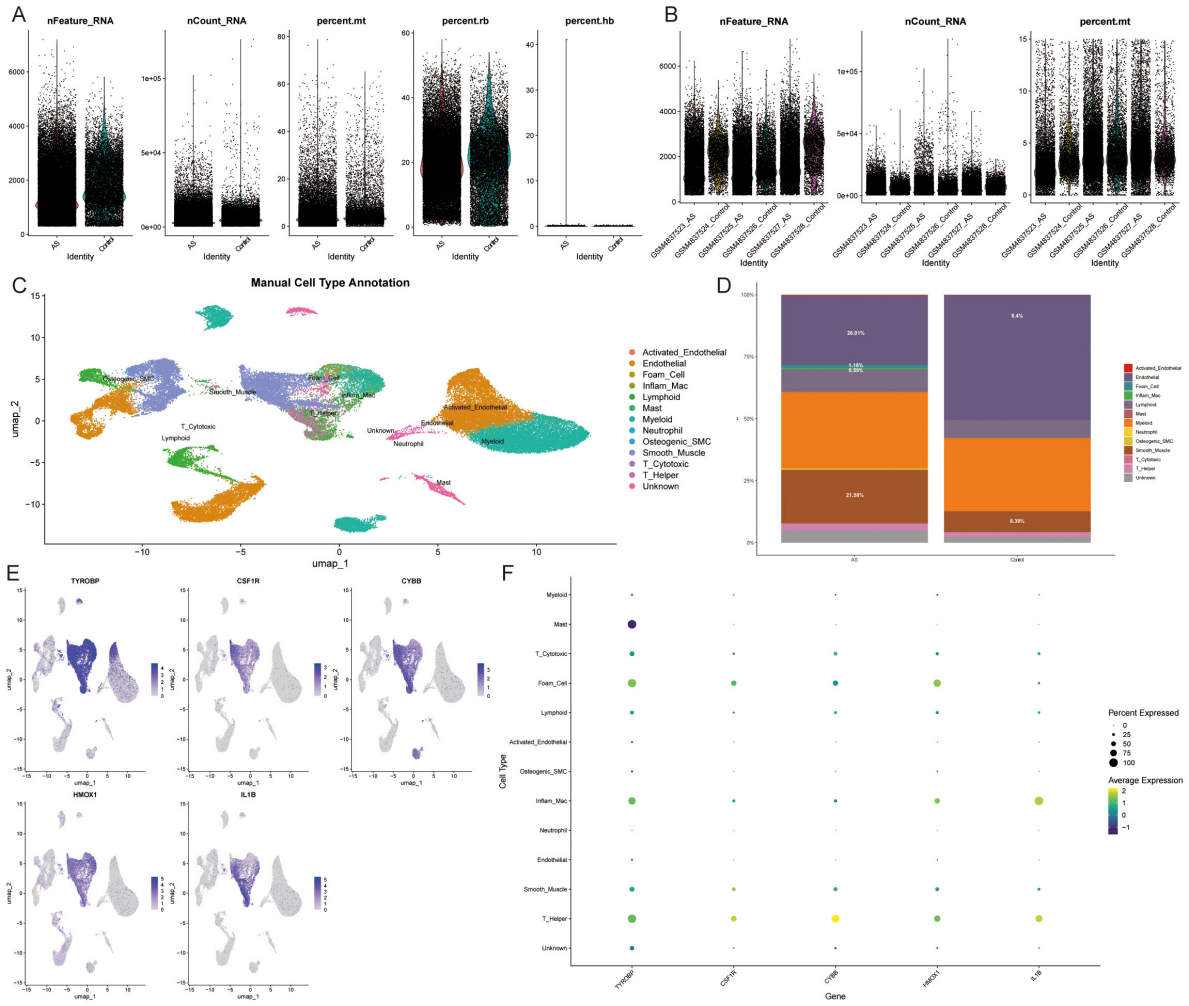
Single-cell data analysis. **(A, B)**. Gene number, sequencing depth, and mitochondrial percentage for different samples. **(C)**. Annotated cell types. **(D)**. Immune cell abundance in AS and control samples. **(E, F)**. Expression of Hub genes in different cell types.

The previous immune infiltration analysis indicated that ASLMRFeGs were significantly associated with M0 macrophages, M1 macrophages, resting mast cells, and resting memory CD4 T cells. We then further analyzed the expression status of ASLMRFeGs and Hub genes in different cells using scRNA-seq datasets. After quality control, normalization, dimensionality reduction with clustering and annotation of the GSE159677 dataset (**Figures 11A, B**), the results likewise showed significant differences in foam cells, inflammatory macrophages, endothelial cells, and smooth muscle cells between AS and control samples (**Figures 11C, D**). In addition, Hub genes were highly expressed in foam cells, inflammatory macrophages, smooth muscle cells, and helper T cells (**Figures 11E, F**).

### 3.5 Gene-drug interaction

Inputting 3 ASLMRFeGs (CYBB, HMOX1, and IL1B) and 2 Hub genes (TYROBP and CSF1R) into the DGIbd yielded 119 potential drugs, of which 57 were FDA-approved and 31 had an “interaction score” > 0.1. Subsequently, through additional literature screening, 13 drugs with potential effects on AS were identified, including Chrysin, Apigenin, Vitamin D, Selenium, Sunitinib, Canakinumab, Rilonacept, Diacetylrhein, Tiludronic acid, Risedronic acid, Clodronic acid, Etidronic acid, and Hydroquinone.

To investigate the interaction affinity of the drugs with the Hub gene and the key gene for ferroptosis, GPX4, we employed molecular docking techniques. Due to the large molecular weights of Canakinumab and Rilonacept, and the limitations imposed by Selenium as a metal molecule in accessing their 3D structure data, we were unable to include these compounds in our analysis and had to exclude them. Additionally, Vitamin D was separated into its two forms, D2 and D3, for individual assessment. The molecular docking analysis involved 11 drugs and 6 proteins: IL1B (1L2H), HMOX1 (1N45), CSF1R (2I0V), CYBB (3A1F), GPX4 (2OBI), and TYROBP (2L34). Out of 66 docking interactions, 51 showed a pooled energy of ≤ -5.0 kcal/mol, and all drugs demonstrated binding energies to GPX4 of <-5.5 kcal/mol (**Table 1**). These findings suggest a potential association of these drugs with the process of ferroptosis.

**Table 1:** The binding energy of the drug and the Hub genes. (kcal/mol).

Drug	CSF1R	CYBB	GPX4	HMOX1	IL1B	TYROBP
Hydroquinone	-6.4	-5.1	-5.7	-6.1	-5.0	-4.2
Etidronic acid	-6.4	-5.0	-5.7	-6.1	-5.0	-4.2
Risedronic acid	-6.4	-5.1	-5.6	-5.6	-5.1	-4.2
Clodronic acid	-6.4	-5.1	-5.7	-6.2	-4.8	-4.3
Diacetylrhein	-6.4	-5.0	-5.6	-6.1	-5.5	-4.4
Tiludronic acid	-6.3	-5.0	-5.7	-6.2	-5.0	-4.2
Apigenin	-6.3	-5.0	-5.6	-5.9	-5.2	-4.2
Vitamin D2	-6.4	-5.1	-5.7	-5.9	-4.7	-4.4
Vitamin D3	-6.4	-5.0	-5.6	-6.1	-4.8	-4.4
Chrysin	-6.3	-5.0	-5.5	-6.1	-5.0	-4.4
Sunitinib	-6.3	-5.0	-5.7	-5.8	-4.7	-4.1

## 4 Discussion

Ferroptosis has emerged as a critical process implicated in the development of AS, suggesting that targeting ferroptosis could hold promise as a therapeutic strategy for CVDs (27). Moreover, lipid metabolism plays an important role in the regulation of cell survival and death, which may affect cellular susceptibility to ferroptosis by influencing the processes of lipid synthesis, degradation, storage, conversion, and utilization (28). Therefore, lipid metabolism, serving as an intermediary process, intricately connects AS and ferroptosis. Prior research has shown that improving lipid metabolism can effectively delay AS progression by enhancing mitochondrial function, reducing oxidative stress and inflammation, and inhibiting ferroptosis (27, 29). To gain further insights into the underlying mechanisms of lipid metabolism in the development of ferroptosis in AS, this study employed bioinformatics analysis to investigate ASLMRFeGs, subtype AS samples based on the ASLMRFeGs, and develop a prediction model using machine learning techniques, aiming to elucidate the potential associations between lipid metabolism and ferroptosis in AS.

We validated this using two cell lines: ox-LDL-induced HUVECs and RAW264.7 cells to construct *in vitro* models of endothelial damage and foam cells due to abnormal lipid metabolism. Our goal was to demonstrate that abnormal lipid metabolism causes endothelial cells and macrophages to undergo ferroptosis, which accelerates the formation of AS. The experiments showed that the MOD group exhibited significant lipid accumulation and massive ferroptosis in endothelial cells and macrophages, which was ameliorated by the addition of Fer-1. This suggests that aberrant lipid metabolism induces ferroptosis in endothelial cells and macrophages, thereby promoting AS. Our further studies found that abnormal lipid metabolism causes dysfunction or abnormal activation of various organelles, which may also be the mechanisms for the occurrence of ferroptosis in AS. Specifically, mitochondrial and lysosomal dysfunction, as well as ERS, were identified as key contributors to ferroptosis in this context.

### 4.1 The role of ASLMRFeGs in AS and ferroptosis

The initial step involved differential expression analysis to compare gene expression levels between control and AS samples. The analysis identified CTSB, CYBB, DPP4, HMOX1, and IL1B as highly expressed in AS, whereas HILPDA exhibited lower expression levels. External validation and *in vitro* experiments demonstrated that CYBB, HMOX1, and IL1B were upregulated in endothelial injury and foam cell models.

The CYBB gene encodes NADPH oxidase 2 (NOX2), a pivotal enzyme responsible for the generation of ROS within the vascular system (30). ROS play a critical role in the pathogenesis of AS through multiple mechanisms, including the modulation of cellular proliferation and death, the regulation of inflammatory responses, the induction of oxidative stress, the promotion of lipid peroxidation, the impairment of endothelial function, and the dysregulation of vascular tone (31, 32). NOX2 is primarily expressed in macrophages, and its mRNA expression level demonstrates a strong correlation with the severity of AS lesions (33). Clinical evidence indicates that genetic deficiency of NOX2 significantly attenuates atherosclerotic burden (34). Furthermore, NOX2-specific inhibitors have shown therapeutic potential in AS by stabilizing vulnerable plaques through enhanced macrophage efferocytosis via the MertK/PI3K/AKT pathway (35), and by retarding AS progression through the mitigation of oxidative stress and suppression of angiogenic factors, including VEGF and HIF-1α (36). Therefore, the CYBB and its encoded NOX2 enzyme hold significant pathophysiological importance in AS and are anticipated to serve as potential targets for the diagnosis and treatment of AS.

Heme oxygenase-1 (HMOX1) is a critical enzyme that degrades heme into carbon monoxide, biliverdin, and ferrous ions. In AS lesions, HMOX1 expression is significantly upregulated in foamy macrophages, yet its role in AS pathogenesis is complex and context-dependent (37, 38). On the one hand, HMOX1 promotes AS progression by interacting with lactate dehydrogenase B to facilitate the degradation of mitochondrial transcription factor A by Lon peptidase 1, which leads to mitochondrial dysfunction and ferroptosis, thereby exacerbating AS (39). Moreover, overexpression of HMOX1 in macrophages drives inflammation and ferroptosis-related oxidative stress, increasing plaque burden in AS mouse models (40). However, on the other hand, genetic deletion of HMOX1 worsens AS lesion progression in LDLR-deficient mice, suggesting a protective role. This protective effect may be mediated by HMOX1’s antioxidant properties, suppression of lipid peroxidation, and modulation of nitric oxide pathways (41, 42).

Interleukin-1β (IL-1β) is a potent pro-inflammatory cytokine that plays a key role in the pathogenesis of AS. Compared to the normal population, AS patients exhibit significantly elevated mRNA and protein levels of IL-1β, which are positively correlated with disease severity (43, 44). In the early stages of AS, IL-1β promotes inflammatory responses by inducing the expression of endothelial cell adhesion factors (e.g., ICAM-1, VCAM-1) and chemokines (e.g., MCP-1), which in turn drive the accumulation of inflammatory cells in the vasculature as well as invasion into the intima (45). In recent years, several new drug studies and clinical trials have demonstrated that inhibition of IL-1β effectively reduces the risk of residual inflammation in atherosclerotic cardiovascular disease, targeting IL-1β as a potential therapeutic target for AS (46–48). However, while the role of IL-1β in the inflammatory response has been extensively studied, the specific mechanisms by which IL-1β affects AS through modulation of ferroptosis remain unclear. A recent study revealed that IL-1β can regulate iron-sulfur cluster homeostasis by inducing the acetylation of the mitochondrial inner membrane protein nicotinamide nucleotide transhydrogenase, thereby inhibiting ferroptosis in tumor cells and mediating immunotherapy resistance (49). This finding suggests that IL-1β may similarly influence cellular iron metabolism and ferroptosis processes in AS, thereby affecting disease progression.

### 4.2 Lipid metabolism influences ferroptosis by regulating organelle function

WGCNA identified a total of 225 module Hub genes associated with atherosclerotic lipid metabolism and ferroptosis. Functional enrichment analysis revealed that these genes are involved in crucial biological processes, including inflammation, immune response, cytoskeleton organization, and cell migration, which have been extensively studied and confirmed in AS (50–52). In addition, these module Hub genes also exhibit close associations with various organelles, such as mitochondria, lysosomes, the ER, and the Golgi apparatus. Therefore, we investigated the role of these organelles in ferroptosis in the AS lipid metabolism pathway. The experimental results showed that abnormal lipid metabolism damaged mitochondrial and lysosomal function and promoted ERS, which in turn promotes ferroptosis. Inhibition of ferroptosis using Fer-1 antagonizes the damaging effects of lipid metabolism abnormalities.

#### 4.2.1 Mitochondrial dysfunction and ferroptosis

Our experiments demonstrated that abnormal lipid metabolism can damage mitochondrial mtDNA, causing mitochondrial dysfunction, promoting ROS production, and increasing mitochondrial Fe^2+^ content, which in turn promotes ferroptosis. Mitochondria, as a major source of ROS in cells, play a crucial role in regulating cellular metabolism, signal transduction, and death signaling, especially in iron metabolism as well as material and energy metabolism (53). Mitochondrial redox function plays a decisive role in the development of AS. By enhancing mitochondrial oxidative metabolism promotes fatty acid degradation, reduces intracellular lipid accumulation, limits foam cell formation, and delays the onset of AS (54, 55), whereas inhibition of ferroptosis can attenuate AS by decreasing lipid peroxidation and endothelial dysfunction (56). Studies have revealed distinct changes in mitochondria during ferroptosis, including marked shrinkage, increased membrane density, and a reduction or disappearance of mitochondrial cristae, which is not consistent with characteristics of other cell death, such as apoptosis and cell necrosis (57). To counteract the detrimental effects of ferroptosis on mitochondria, mitochondria significantly inhibit oxidative stress-induced ferroptosis by overexpressing mitochondrial ferritin or mitochondrial catalase, which inhibit mitochondrial iron overload or ROS accumulation (58). In addition, mitochondria play a central role in fatty acid metabolism by providing specific lipid precursors for lipid oxidation, which is a key step in cellular ferroptosis (53). Mitochondrial energy metabolism is also closely intertwined with ferroptosis, and inhibition of the mitochondrial tricarboxylic acid cycle or the electron transfer chain can attenuate mitochondrial membrane potential hyperpolarization, lipid peroxide accumulation, and ferroptosis (59).

#### 4.2.2 Lysosomal dysfunction and ferroptosis

Lysosomes, as acidic membrane-bound organelles, serve as the endpoints of various vesicular transport pathways, including endocytosis, phagocytosis, and autophagy pathways, which are essential for promoting lipid catabolism and transport and maintaining cellular lipid homeostasis (60). Lysosomal proteins LAMP1 and LAMP2 have been shown to bind cholesterol with high affinity and specificity through their luminal domain 1 and facilitate the flow of cholesterol through the NPC2-NPC1 export pathway (61). Additionally, lysosomes may rely on membrane contacts with other organelles for lipid transfer, and cholesterol transfer in the ER-to-lysosomal direction was enhanced in a VAP-dependent manner by overexpression of STARD-3 (62); STARD-3 also transfers cholesterol from lysosomes to mitochondria to provide precursors for steroid hormones synthesized in mitochondria (63).

Recently, it was found that inhibition of lysosome-dependent cell death limited Erastin-induced ferroptosis, suggesting that ferroptosis is a lysosomal cell death process and that lysosomes contribute to ferroptosis through mechanisms such as autophagy activation, the release of lysosomal cathepsins, and the accumulation of lysosomal iron or nitric oxide (64, 65). Many studies have shown that excessive activation of autophagy and lysosomes degrades ferritin, increases unstable iron accumulation, and promotes iron-dependent cell death (66). Cathepsin B (CTSB) is a mediator of organelle-specific initiation of ferroptosis, and activation of the transcription factor STAT3 upregulates CTSB, which is translocated from the lysosome to the nucleus, leading to DNA damage and subsequent STING1-dependent ferroptosis (65). Conversely, CTSB-dependent albumin catabolism promotes glutathione synthesis by exporting cystine from lysosomes via the transporter cystinosis fuels, thereby inhibiting lethal lipid peroxidation (67). Additionally, it was found that lysosomal damage caused by mitochondrial dysfunction induced ferroptosis (68). This indicates that lysosomes regulate lipid metabolism through membrane contacts with other organelles, influencing autophagy, cathepsin release, and thus ferroptosis. Our study suggests that endothelial cell injury and foam cell models may lead to activation of lysosomes and promotion of autophagy in the initial phase, followed by lysosomal over-excitation, causing dysfunction, whereas inhibition of ferroptosis improves lysosomal function.

#### 4.2.3 ERS and ferroptosis

The ER plays a critical role in the synthesis, processing, packaging, and transportation of proteins and lipids. When unfolded or misfolded proteins accumulate in the ER, it leads to a pathological condition known as ERS. To counter ERS, cells initiate the unfolded protein response (UPR) signaling cascade by activating the expression of molecular chaperones, regulating lipid synthesis, and promoting ER-associated degradation, which helps reduce the levels of unfolded or misfolded proteins and restore ER homeostasis (69, 70). ERS has been identified as a key regulator of cholesterol deposition, macrophage differentiation, and endothelial dysfunction. The ER is directly involved in the formation of lipid droplets and the maintenance of lipid homeostasis. When disorders of lipid metabolism are exacerbated, it promotes ERS and URP, disrupting the normal metabolism of adipose tissue, triggering an inflammatory response in adipocytes, enhancing the secretion of adipokines, and causing ectopic lipid deposition, which contributes to the development of metabolic diseases (71). This situation is particularly pronounced in obese patients, where overnutrition stimulates adipogenesis, creating an imbalance in the body’s microenvironment, inducing progressive hypertrophy of adipocytes, stimulating the ER to synthesize more proteins for the formation of lipid droplets, and altering the flow and thickness of ER membranes via the ER sensors IRE1α and PERK, which lead directly to ERS (72). Furthermore, ERS is an important cause of endothelial and macrophage apoptosis in advanced lesions (73). Inhibiting macrophage ERS promotes the polarization of differentiated M2 macrophages toward an M1 phenotype, thereby inhibiting foam cell formation (74). ERS also contributes to an imbalance between nitric oxide and ROS, causing oxidative stress, damaging endothelial cells, and promoting the progression of AS and plaque formation (73, 75).

The classical UPR signaling pathway involves the activation of three classes of ER membrane proteins: IRE1, PERK, and ATF6 (71). In normal conditions, the chaperone protein Bip and GRP78 bind to these three proteins, preventing their activation and keeping them in an inactive state. However, when external stimuli or changes in ER homeostasis result in the excessive accumulation of misfolded or unfolded proteins, Bip binds to the abnormal proteins in the ER lumen, dissociates from and activates IRE1α, PERK, and ATF6 to carry out their respective functions. This activation enhances ER protein folding capacity, inhibits intracellular protein synthesis, and restores ER homeostasis (76). Nevertheless, if the degree of ERS surpasses the UPR’s regulatory range, it disrupts ER homeostasis, leading to various metabolic abnormalities associated with ER function. GRP78 and CHOP are commonly used as markers of ERS, and our experiments also validated the three branches of ERS. First, based on the expression of GRP78 and CHOP genes, it was shown that abnormal lipid metabolism significantly activated ERS, which might be excessive, and the use of Fer-1 inhibited the excessive ERS of the cells. It was further found that the abnormal lipid metabolism regulated the UPR branch of IRE1 and PERK, and the use of Fer-1 regulated the branches of IRE1, PERK, and ATF6.

In the first branch, autophosphorylated IRE1 activates the RNase structural domain and catalyzes the splicing of XBP1 mRNA, generating the active transcription factor XBP-1s. XBP-1s is involved in inflammation, cell survival, lipid metabolism, and calcification to regulate endothelial cell proliferation, transformation, and apoptosis, smooth muscle cell phenotypic switching, and the accumulation of foam cells that thereby affect AS (77). Furthermore, the overexpression of IREI and XBP1 has been shown to increase cellular susceptibility to ferroptosis (78, 79). Additionally, IRE1 activates c-Jun NH2 terminal kinase (JNK) in response to ERS. Inhibition of the IRE1/JNK pathway not only reduces ERS-induced apoptosis and improves vascular endothelial dysfunction (80) but also attenuates ferroptosis in acute kidney injury (81). Collectively, these studies indicate the IRE1 branch plays a crucial role in AS progression through XBP-1s/JNK signaling, ERS-induced apoptosis, and enhanced ferroptosis susceptibility. Unexpectedly, our experiments revealed that ox-LDL-induced lipid accumulation promotes ERS while concomitantly downregulating XBP1 expression. This apparent paradox, ERS induction with reduced IRE1/XBP1 signaling, suggests a potential compensatory adaptation may occur under sustained lipid stress. We hypothesize that persistent lipid overload triggers feedback inhibition of the IRE1/XBP1 pathway to mitigate excessive cellular stress responses, which could consequently reduce ferroptosis susceptibility. This interpretation aligns with our observation that ferroptosis inhibition (Fer-1) alleviates both lipid accumulation and ERS severity. Under such conditions of reduced lipid stress, restoring IRE1/XBP1 signaling could theoretically help reestablish ER homeostasis through ER-associated degradation of misfolded proteins and upregulation of pro-homeostatic genes (77), potentially delaying AS progression. However, the precise mechanisms underlying lipid-mediated suppression of IRE1/XBP1 and its functional consequences require further experimental investigation.

In the second branch, PERK plays a dual role, serving not only to reduce the protein load of ERS but also to signal the cell death pathway. Phosphorylated PERK phosphorylates eIF2α, thereby inhibiting mRNA translation, reducing protein influx into the ER, and facilitating the translation of ATF4. CHOP is an important target gene regulated by ATF4, and sustained ERS causes CHOP to activate genes responsible for encoding apoptosis (82). In addition, PERK acts as a negative regulator of ferroptosis, and the occurrence of ferroptosis is accompanied by the activation of the PERK-eIF2α-ATF4-CHOP signaling pathway, and inhibition of ERS reduces ferroptosis (83–85). Our experiments found that abnormal lipid metabolism up-regulated the expression of the EIF2A and CHOP genes; the use of Fer-1 improved the expression of the PERK, EIF2A, and CHOP genes, possibly due to the negative feedback regulation of up-regulated EIF2A to inhibit the expression of PERK.

In the third branch, ATF6 is released and translocated to the Golgi, where it undergoes cleavage by resident proteases (site 1 and site 2 proteases) and subsequently moves to the nucleus to activate the transcription of various chaperone molecules. Previous studies have shown that increased expression of ATF6α contributes to enhanced resistance against ferroptosis (86).

#### 4.2.4 Golgi apparatus and ferroptosis

Additionally, the Golgi apparatus plays a key role in regulating protein and lipid synthesis, modification, and distribution within the cell. Golgi stress not only triggers the production of ROS but also promotes the accumulation of lipid peroxides, ultimately inducing ferroptosis (87). In short, the module genes obtained by WGCNA provide a preliminary indication of the mechanisms by which abnormalities of lipid metabolism regulate AS and ferroptosis, in which inflammation and immune responses play key roles. In particular, the abnormalities of lipid metabolism may affect ferroptosis and AS by regulating cell organelle function.

### 4.3 Machine learning models identification of hub genes

Our integrated bioinformatics and machine learning pipeline (LASSO, SVM-RFE, and RF) robustly identified four Hub genes (TYROBP, CSF1R, LCP2, C1QA) significantly associated with AS lipid metabolism and ferroptosis pathways based on the GSE100927 transcriptomic dataset. However, subsequent *in vitro* validation in ox-LDL-induced models yielded crucial and unexpected findings that necessitated refinement of the final predictive model. Bioinformatics analysis indicated upregulation of C1QA in AS samples. However, our *in vitro* experiments in both HUVECs and RAW 264.7 cells consistently showed that ox-LDL treatment downregulated C1QA expression. Furthermore, ferroptosis inhibition (Fer-1) did not significantly rescue this downregulation. This stark contrast between the bioinformatics prediction and experimental observation suggests that C1QA’s regulation in human AS tissue might involve complex microenvironmental factors, compensatory mechanisms, or cell-type interactions not fully recapitulated in the simplified *in vitro* model, or potentially reflect differences in disease stage. While LCP2 was upregulated by ox-LDL *in vitro* (aligning directionally with its bioinformatics association), its response to ferroptosis inhibition was cell-type specific: Fer-1 significantly attenuated LCP2 upregulation only in RAW 264.7 macrophages, not in HUVECs. This suggests LCP2’s role in AS ferroptosis may be more prominent within the myeloid compartment, a nuance masked in bulk tissue analysis.

This discrepancy highlights a fundamental strength and limitation of purely computational biomarker discovery. Machine learning excels at identifying correlative signatures from complex bulk tissue data but cannot inherently predict. Discordance between *in vivo* (bioinformatics) and *in vitro* experimental gene regulation. Whether a gene’s dysregulation is mechanistically linked to a specific pathway (ferroptosis) in all relevant cell types. Our findings underscore that experimental validation is essential not only to confirm dysregulation but also to assess directionality, pathway linkage, and cell-type specificity within the specific pathological context being modeled. The *in vitro* model revealed crucial nuances masked in the bulk analysis.

Given the contradictory directionality for C1QA and the inconsistent ferroptosis-linkage of LCP2 across cell types, we prioritized genes for the nomogram based on concordant bioinformatics association AND consistent experimental validation in our model system. TYROBP and CSF1R met these criteria, which were identified by machine learning, showed concordant upregulation *in vitro*, and their upregulation was consistently attenuated by ferroptosis inhibition in both cell lines. Consequently, the final nomogram included TYROBP and CSF1R. However, the exclusion of LCP2 and C1QA does not diminish the validity of the machine learning approach but rather exemplifies its role as a discovery engine that requires biological grounding. They emphasize that experimental validation is indispensable for translating computational predictions into biologically relevant biomarkers and understanding their mechanistic drivers; bulk transcriptomics has limitations in resolving cell-type specific responses and differentiating primary drivers from secondary effects. Additionally, the C1QA contradiction suggests its role in AS may be highly context-dependent or involve mechanisms not captured by acute ox-LDL exposure in single cell types, warranting investigation into complement signaling dynamics in AS ferroptosis; the LCP2 cell-specificity reinforces the central role of macrophages in ferroptosis-related processes within AS plaques. Future studies employing single-cell or spatial transcriptomics on AS lesions, coupled with targeted *in vivo* modulation, will be crucial to resolve these discrepancies, understand the cell-specific roles of LCP2 and C1QA, and further validate the predictive power of TYROBP and CSF1R.

TYROBP, a protein tyrosine kinase-binding protein, is widely expressed in natural killer cells, neutrophils, and monocytes/macrophages. Studies have shown that TYROBP/DAP12 is highly expressed in the plaques of high-fat diet-fed ApoE^-/-^ mice, where it promotes AS formation through the TREM-1/DAP12 pathway. This effect can be mitigated by pravastatin, which modifies the inhibition of this pathway (88). Similarly, TYROBP has been found to be highly expressed in the aorta of a high-fat diet-fed novel Tibetan minipig model of AS (89). Multiple bioinformatics analyses have also identified TYROBP as upregulated in AS, suggesting its potential as a biomarker for the AS (90–92). Although the relationship between TYROBP and ferroptosis has not yet been fully elucidated, a mouse model of lipopolysaccharide-induced acute lung injury revealed that TREM2 can inhibit DAP12 expression and reduce ferritin accumulation, thereby inhibiting macrophage ferroptosis (93).

CSF1R, a colony-stimulating factor 1 receptor, is highly expressed in macrophages and regulates their survival, proliferation, and function by binding to its ligand, CSF1 (94). Inhibition of CSF1R has been shown to suppress macrophage proliferation, thereby slowing the progression of AS (95). Conversely, activation of CSF1R promotes lipid uptake by macrophages and the formation of foam cells, a critical step in the development of atherosclerotic plaques (96). Moreover, CSF1R activation is closely associated with inflammatory responses. For instance, IL-6 enhances STAT3 activity, which further upregulates CSF1R expression and boosts macrophage survival and secretion of inflammatory factors, thereby exacerbating AS (97). Additionally, GRK5 has been reported to reduce AS by desensitizing macrophage CSF1R (98).The relationship between CSF1R and ferroptosis is not yet fully understood. However, CSF1R has been identified by Xu et al. as a potential biomarker associated with ferroptosis in AS and atrial fibrillation (99).

### 4.4 Clinical implications and future directions

Then, we selected three ASLMRFeGs (CYBB, HMOX1, and IL1B) and two Hub genes (TYROBP and CSF1R) of the models combined with the results of the *in vitro* experiments and constructed a nomogram to predict the incidence of AS, which was validated in three external datasets and demonstrated satisfactory predictive ability to effectively differentiate between AS and normal samples.

In the above analysis, we obtained three ASLMRFeGs and two Hub genes, which we believe are also potential targets for the treatment of AS. Therefore, we obtained 13 potential drugs for the treatment of AS by querying databases and literature. Chrysin is a flavonoid compound, and its protective effects on the cardiovascular system have been confirmed. It can delay AS by improving lipid metabolism, enhancing vascular function, and inhibiting inflammatory responses (100). Apigenin is also a natural flavonoid compound. Thanks to its antioxidant and anti-inflammatory properties, as well as its antihypertensive effects and regulation of lipid metabolism, it is considered a candidate drug for treating AS (101). Vitamin D is a fat-soluble vitamin. Studies have found that vitamin D deficiency is associated with the development and progression of AS, and it increases the risk of severe coronary artery disease in women (102). Supplementation with vitamin D can reduce the formation of VSMC foam cells through the JNK-TLR4 signaling pathway, thereby delaying the progression of AS (103). Selenium is an essential trace element for the human body. It is absorbed and metabolized into selenocysteine to produce selenoproteins. Due to their antioxidant properties, selenoproteins show certain therapeutic potential in AS. Studies have shown that selenium supplementation can eliminate AS, improve plaque vulnerability, and enhance vascular tension (104). Sunitinib is primarily used for treating tumors and was previously thought to have certain cardiotoxicity (105, 106). However, recent findings indicate that due to its potent anti-inflammatory characteristics, it exhibits immunomodulatory effects in chronic cardiovascular inflammation models, reducing circulating TNF-α levels, and is thus considered a potential drug for inflammatory diseases (107).Canakinumab and Rilonacept are IL-1β antagonists. Targeting inflammatory pathways for AS treatment is considered an important approach to reducing residual inflammatory risk in atherosclerotic cardiovascular disease (108–110). Diacetylrhein is an orally active anthraquinone compound that mainly inhibits the activation of IL-1β by reducing the production of IL-1 converting enzyme. It has been found to alleviate AS caused by IL-1 (111). Tiludronic acid, risedronic acid, clodronic acid, and etidronic acid are all bisphosphonates, which are drugs used to treat hypercalcaemia and osteoporosis. However, numerous reports have found that they have certain therapeutic potential for AS (112). A meta-analysis found that nitrogen-containing bisphosphonates can reduce the intima-media thickness and plaque area in AS patients (113). Hydroquinone is a phenolic compound widely found in nature and has antioxidant properties (114). Its various derivatives are considered potential drugs for treating AS due to their anti-inflammatory, antioxidant, and endothelial function-improving effects (115–117).

Through database and literature searches, we identified several potential drugs for the treatment of AS. Our discovery was based solely on literature that suggests these drugs may have therapeutic and ameliorative effects on AS. However, it is not clear whether these drugs can treat AS by improving lipid metabolism and regulating ferroptosis. Therefore, we utilized molecular docking techniques and found that these drugs exhibit good binding affinity with the key ferroptosis gene GPX4, suggesting the possibility of their regulation of ferroptosis. Nevertheless, these findings still require further experimental validation.

### 4.5 Innovations and limitations

In this study, a comprehensive bioinformatics analysis was conducted to investigate the potential mechanisms linking ferroptosis in lipid metabolism pathways to AS. Three ASLMRFeGs (CYBB, HMOX1, and IL1B) were identified, highlighting the close association between ferroptosis mediated by lipid metabolic pathways and immune responses, as well as its impact on the immune infiltration microenvironment in AS. Furthermore, two distinct lipid metabolism pathway ferroptosis-related molecular clusters were discovered in AS samples, exhibiting significant disparities in immune response and inflammation. Machine learning models and *in vitro* experiments identified two Hub genes (TYROBP and CSF1R), and their roles in AS pathogenesis and progression were delineated. Moreover, a nomogram was constructed using Hub genes combined with ASLMRFeGs to predict the risk of AS development, demonstrating promising diagnostic efficacy. Additionally, this study identified 13 potential drugs for the treatment of AS based on ASLMRFeGs and Hub genes. Finally, we induced HUVECs and RAW 264.7 cells using ox-LDL in an attempt to establish a model of endothelial damage and foam cells caused by abnormal lipid metabolism. It was demonstrated that lipid metabolism abnormalities prominently contributed to ferroptosis and that inhibition of ferroptosis improved the expression of CYBB, HMOX1, IL1B, TYROBP, and CSF1R genes, which are important genes for lipid metabolism to regulate ferroptosis and AS.

Ferroptosis, a novel form of cell death, has been the subject of numerous bioinformatics studies exploring its link to AS. However, the pathogenesis of AS, intricately tied to lipid metabolism abnormalities and immune dysregulation, presents a research gap concerning the bioinformatics of ferroptosis induced by lipid metabolism disturbances within the atherosclerotic context, a gap our study begins to bridge. Subsequently, while the association of the five hub genes we identified, CYBB, HMOX1, IL1B, TYROBP, and CSF1R, with AS has been confirmed by various studies, the relationship of these genes with lipid metabolism and ferroptosis, particularly in the atherosclerotic context, remains unclear. This is especially true for IL1B, TYROBP, and CSF1R, warranting further investigation. Additionally, through the validation of a dual-cell line model, we discovered various organelle dysfunctions associated with the interplay between abnormal lipid metabolism and ferroptosis.

Nevertheless, it should be noted that although we used a two-cell line for our experiments, the *in vitro* model does not perfectly replicate the intricate regulatory mechanisms in the human body. Further confirmation of the specific expression patterns of these genes in animal models of AS and in humans is necessary, and additional investigations are required to unravel their precise mechanisms. Furthermore, our study has uncovered potential mechanisms for the treatment of AS. However, additional clinical studies are necessary to validate the efficacy of these drugs in managing AS. Moreover, further experimental validation is required to ascertain whether these medications exert their therapeutic effects on AS by modulating ferroptosis and lipid metabolism.

The multifaceted approach employed in this study, encompassing bioinformatics analysis, molecular characterization, and machine learning modeling, advances our understanding of AS pathophysiology. It provides a foundation for the development of personalized diagnostic tools and innovative therapeutic strategies to combat this prevalent and challenging cardiovascular condition. Inflammatory and immune responses have emerged as potential mechanisms underlying ferroptosis in AS lipid metabolism pathways, shedding light on the intricate interplay between lipid metabolism, ferroptosis, and immune dysregulation in AS. In summary, our study offers a novel perspective on understanding AS, which may guide future research endeavors in this field.

## 5 Conclusions

CYBB, HMOX1, IL1B, TYROBP, and CSF1R are key genes associated with ferroptosis, a form of cell death triggered by lipid metabolism abnormalities in the context of atherosclerosis. These genes are also crucial in modulating the immune-infiltrated microenvironment in patients with AS. Inflammatory and immune responses may serve as pivotal mechanisms through which ferroptosis manifests within atherosclerosis lipid metabolism pathways. Abnormal lipid metabolism promotes ferroptosis and may contribute to the progression of AS, potentially through the modulation of organelle function.

## Data Availability

The original contributions presented in the study are included in the article/**Supplementary Material**. Further inquiries can be directed to the corresponding authors.

## Glossary

AS: Atherosclerosis
ASLMRFeGs: Atherosclerosis Lipid Metabolism-Related Ferroptosis Genes
CCK-8: Cell Count Kit-8
CDF: Cumulative Distribution Function
CVDs: Cardiovascular Diseases
DCA: Decision Curve Analysis
DEGs: Differentially Expressed Genes
ER: Endoplasmic Reticulum
ERS: Endoplasmic Reticulum Stress
Fer-1: Ferrostatin-1
FeRGs: Ferroptosis-Related Genes
GEO: Gene Expression Omnibus
GSEA: Gene Set Enrichment Analysis
GSVA: Gene Set Variation Analysis
HUVECs: Human Umbilical Vein Endothelial Cells
KEGG: Kyoto Encyclopedia of Genes and Genomes
LASSO: Least Absolute Shrinkage and Selection Operator
LMRGs: Lipid Metabolism-Related Genes
NOX2: NADPH oxidase 2
ORO: Oil Red O
ox-LDL: Oxidized Low-Density Lipoprotein
PPI: Protein-Protein Interaction
qRT-PCR: Quantitative Real-Time Polymerase Chain Reaction
RF: Random Forest
ROC: Receiver Operating Characteristic
ROS: Reactive Oxygen Species
SVM-RFE: Support Vector Machine-Recursive Feature Elimination
UPR: Unfolded Protein Response
WGCNA: Weighted Gene Co-expression Network Analysis
